# Decreasing Proportion of Recent Infections among Newly Diagnosed HIV-1 Cases in Switzerland, 2008 to 2013 Based on Line-Immunoassay-Based Algorithms

**DOI:** 10.1371/journal.pone.0131828

**Published:** 2015-07-31

**Authors:** Jörg Schüpbach, Christoph Niederhauser, Sabine Yerly, Stephan Regenass, Meri Gorgievski, Vincent Aubert, Diana Ciardo, Thomas Klimkait, Günter Dollenmaier, Corinne Andreutti, Gladys Martinetti, Marcel Brandenberger, Martin D. Gebhardt

**Affiliations:** 1 University of Zurich, Institute of Medical Virology, Swiss National Center for Retroviruses, Winterthurerstrasse 190, CH-8057, Zurich, Switzerland; 2 Blood Transfusion Service, Swiss Red Cross Berne, Berne, Switzerland; 3 Geneva University Hospitals, Laboratory of Virology, Genève, 14, Switzerland; 4 University Hospital, Clinic for Immunology, Zurich, Switzerland; 5 University of Berne, Institute of Infectious Diseases, Berne, Switzerland; 6 University Hospital, Service of Immunology and Allergy, University Hospital Center, Lausanne, Switzerland; 7 Institut Dr. Viollier AG, Basel, Switzerland; 8 University of Basel, Institute for Medical Microbiology, Basel, Switzerland; 9 Zentrum für Labormedizin St. Gallen, St. Gallen, Switzerland; 10 Clinique de la Source, Laboratoire, Lausanne, Switzerland; 11 Ente ospedaliero cantonale, Servizio di microbiologia, Bellinzona, Switzerland; 12 Labor Synlab Luzern, Lucerne, Switzerland; 13 Swiss Federal Office of Public Health, CH-3003, Berne, Switzerland; Yale School of Public Health, UNITED STATES

## Abstract

**Background:**

HIV surveillance requires monitoring of new HIV diagnoses and differentiation of incident and older infections. In 2008, Switzerland implemented a system for monitoring incident HIV infections based on the results of a line immunoassay (Inno-Lia) mandatorily conducted for HIV confirmation and type differentiation (HIV-1, HIV-2) of all newly diagnosed patients. Based on this system, we assessed the proportion of incident HIV infection among newly diagnosed cases in Switzerland during 2008-2013.

**Methods and Results:**

Inno-Lia antibody reaction patterns recorded in anonymous HIV notifications to the federal health authority were classified by 10 published algorithms into incident (up to 12 months) or older infections. Utilizing these data, annual incident infection estimates were obtained in two ways, (i) based on the diagnostic performance of the algorithms and utilizing the relationship ‘incident = true incident + false incident’, (ii) based on the window-periods of the algorithms and utilizing the relationship ‘Prevalence = Incidence x Duration’. From 2008—2013, 3’851 HIV notifications were received. Adult HIV-1 infections amounted to 3’809 cases, and 3’636 of them (95.5%) contained Inno-Lia data. Incident infection totals calculated were similar for the performance- and window-based methods, amounting on average to 1’755 (95% confidence interval, 1588—1923) and 1’790 cases (95% CI, 1679—1900), respectively. More than half of these were among men who had sex with men. Both methods showed a continuous decline of annual incident infections 2008—2013, totaling -59.5% and -50.2%, respectively. The decline of incident infections continued even in 2012, when a 15% increase in HIV notifications had been observed. This increase was entirely due to older infections. Overall declines 2008—2013 were of similar extent among the major transmission groups.

**Conclusions:**

Inno-Lia based incident HIV-1 infection surveillance proved useful and reliable. It represents a free, additional public health benefit of the use of this relatively costly test for HIV confirmation and type differentiation.

## Introduction

The prevalence of HIV infection is relatively high in Switzerland. Between 0.3% and 0.5% of the Swiss adult population were infected with HIV in 2012, and there were 16’000–27'000 persons alive with HIV [1]. The epidemic started in the 1970s, and the cases notified to the Swiss Federal Office of Public Health (SFOPH) rose rapidly after HIV screening had been introduced in 1985. Notifications decreased during 1992–2000, but rose again by 25% in 2002. The new diagnoses remained relatively stable during the next few years. Among men who had sex with men (MSM), however, the annual notifications almost doubled between 2004 and 2008, while they decreased in most other transmission groups, particularly among persons with heterosexual transmission (HET). Between 2009 and 2013, the notifications generally decreased for all groups, but with a transient 15% increase in 2012 for both MSM and HET [2]. This renewed increase was an object of great concern to those involved in the HIV prevention campaign.

Case surveillance is important, but not sufficient because, due to the long and variable time from infection to diagnosis, it does not reflect actual patterns of HIV transmission. This is why tests for recent infection (TRIs) were developed. Most of them exploit the fact that the HIV-specific antibody response evolves during the first few months of infection with respect to concentration, proportion of total IgG, isotype, avidity, or target antigen pattern [3–6]. Other TRIs are based on the growing diversity of the genotypic HIV variants in a patient [7–9]. The appropriate use of TRIs has been described by a WHO technical report [10]. Common to serology-based TRIs is the need for conducting a special assay that is not part of the diagnostic routine. Systems for prospective country-wide incident HIV-1 infection monitoring based on TRIs were introduced in France in 2003 and in Great Britain in 2009 [11,12]. In the U.S.A., the HIV incidence between 1977 to 2006 was retrospectively estimated based on extrapolation and extended back-calculation of results of a TRI performed in a 17% sample of patients newly diagnosed in 22 states during 2006 [13].

Switzerland has chosen a different approach for monitoring incident HIV infection. We demonstrated in 2007 that a patient’s antibody reaction in a widely used confirmatory line immunoassay, the Inno-Lia HIV I/II Score assay (Inno-Lia), also provides information on the duration of infection [14]. The Inno-Lia measures antibodies to different HIV antigens in a standardized, semi-quantitative way. As both the pattern and intensity of HIV-specific antibodies evolve during the first months after infection, it is possible to define algorithms (Alg) which, with a certain diagnostic sensitivity and specificity, recognize antibody patterns characteristic of early HIV-1 infection. If the diagnostic sensitivity and specificity of an algorithm are known, which requires prior testing of suitable reference groups of either less or more than 12 months duration of infection, it is possible to estimate the number of incident cases by means of the simple diagnostic rule ‘n_tested incident_ = n_true incident_ + n_false incident_’, whereby true-incident and false-incident are calculated based on pre-determined values for diagnostic sensitivity and specificity [14]. Alternatively, if the window periods of the algorithms are known, it is also possible to use the relationship ‘Prevalence = Incidence x Duration’ [5,15].

In previous work, we have determined the diagnostic sensitivity and specificity of more than 20 different Inno-Lia algorithms for differentiating between HIV-1 infections of less or more than 12 months duration. A large study with patients who had been infected for at least 12 months and were selected to represent all clinical stages and major clades of HIV-1, was undertaken to study the specificity of the incident infection algorithms [16]. Of the 714 patients investigated, only 94 were infected by HIV-1 subtype B, while 620 patients were infected by one of 15 different non-B clades including subtypes A, C, D, F, G, H, J, K, and circulating recombinant forms (CRF) 01_AE, 02_AG, 03_AB, 06_CPX, 12_BF and 13_CPX. The study showed that none of these non-B clades impaired the diagnostic specificity of the method. Although a HIV-1 RNA load below 50 copies/mL significantly reduced the specificity among patients receiving antiretroviral treatment (ART), increasing age was the sole factor which weakly impaired the test specificity in untreated patients [16]. In another study, we assessed the diagnostic performance of the algorithms based on 527 incident and 740 older infections. The ten best-performing algorithms had a mean sensitivity of 59.4% for recognizing infections of up to 12 months duration and a mean specificity of 95.1%. Using these ten algorithms in combination, we identified distinct changes between the incident infection ratios (IIR) of four successive annual cohorts of HIV-1 notifications [17]. In a further study, we determined the window periods of all algorithms and calculated the IIR for the same four annual cohorts, but based on the relationship ‘Prevalence = Incidence x Duration’. The resulting IIRs and their changes were very comparable to those determined based on test performance [18].

In 2007, Switzerland adopted a new testing and data-reporting system for HIV confirmation with the intention to put Inno-Lia based incident infection assessment into countrywide practice. Under the guidance of the Swiss National Center for Retroviruses (SNCR), 11 regional notification labs perform the confirmatory testing which, among other tests, comprises a mandatory Inno-Lia for confirmation of HIV infection and differentiation between HIV-1 and HIV-2. These labs notify all results, including those of the Inno-Lia, by e-mail to the SFOPH. This system has worked very well and, since 2008, has provided prospectively collected and increasingly complete data for a population-based assessment of incident HIV infection.

In the present study, the laboratory notification data collected 2008–2013 were linked with supplemental clinical and epidemiological information notified to the SFOPH by the patients’ attending physicians. The linked data were used for studying the dynamics of the HIV epidemic in different transmission groups. Of special interest was the question whether the sudden increase of HIV notifications observed in 2012 after three years of continuous decline was due to an increase in actual HIV transmissions or rather to a more frequent diagnosis of older infections.

## Methods

### Data origin and ethics statement

The present cross-sectional study investigated data contained in the regular, anonymized HIV notifications prospectively sent to the SFOPH for patients newly diagnosed with HIV infection between Jan 2008 and Dec 2013. There were two types of notifications for each patient: (i) a laboratory notification with the diagnostic test data sent in by one of the 11 HIV notification labs or the SNCR, and (ii) a supplemental clinical notification forwarded by the patient’s attending physician, with epidemiologic and clinical information relevant in the context of HIV infection. No informed consent was needed, since both types of anonymous notifications are imposed by Swiss federal law.

### Organization of HIV confirmation and notification

For a detailed description see S1 File. In brief, as soon as a notification laboratory has firmly established the diagnosis of HIV positivity, it sends the test results and the SFOPH’s supplemental HIV notification form to the patient’s attending physician. The physician completes the form and forwards it to the SFOPH. The notification laboratory enters the test results into a dedicated Excel macro-sheet and mails it electronically to the SFOPH. Thus, at the end, every HIV notification should comprise both the laboratory’s and the physician’s notification, and the two sets of data are linked by the SFOPH.

The HIV notification laboratories are commissioned by the SFOPH and accredited according to the ISO/IEC 17025 standard by the governmental Swiss Accreditation Service SAS http://www.seco.admin.ch/sas/index.html?lang=en. The likewise accredited SNCR has been commissioned since 1985 by the SFOPH to serve as the national HIV reference laboratory.

### Serological differentiation between incident and older HIV-1 infection

Detailed results of the Inno-Lia HIV I/II Score assay (Fujirebio, formerly Innogenetics, Ghent, Belgium) were taken from the laboratory notifications sent to the SFOPH. The Inno-Lia is a CE-marked (conforming with the diagnostic standards of the European Union) line immunoassay that measures antibodies against recombinant proteins or synthetic peptides of HIV-1 or HIV-2, which are coated as 7 lines on a nylon strip (S1 Fig). As each test strip also contains three quantitative internal standards indicating different intensities, a semi-quantitative ranking of the antibody reactions is possible [19,20]. Antibody reaction to each of the 7 HIV antigen bands present on the test strips (sgp120 [including HIV-1 group O peptides], gp41, p31, p24 and p17 of HIV-1, and sgp105 and gp36 of HIV-2) was assessed either visually or by the automated scanner–based LIRAS system (Fujirebio, formerly Innogenetics). Based on the three internal standards, which define reaction levels of +/−, 1+, and 3+ for each test strip, the antibody reaction to each HIV antigen was classified into one of six possible intensity scores (-, ±, 1+, 2+, 3+, 4+) either visually according to instructions in the manufacturer’s test kit insert or by the manufacturer’s automated LIRAS reading system. In the laboratory notification, these values are transferred to numerical values (0, 0.5, 1, 2, 3, or 4). For the present study, only the antibody reaction to HIV-1 was of relevance. Therefore, each patient’s pattern of antibodies to the five HIV-1 antigens gp120, gp41, p31, p24 and p17 was subjected to analysis by each of 10 different Inno-Lia incident infection algorithms (see below). Previous investigation has shown that the mode of Inno-Lia evaluation (visual or automated) does not affect the incident infection result [16].

### Inno-Lia incident infection algorithms

Twenty-six algorithms (Alg) for incident HIV-1 infection, all described in [17], were developed empirically by investigating which Inno-Lia antibody patterns were found at maximal frequency in a group of patients with less than 12 months duration of infection (= recent or incident infection) and at minimal frequency in a group of patients with ≥12 months duration of infection (= older infection), as described in detail earlier [14,16,17]. The 10 best-performing algorithms, listed in S1 Table, were applied to the collected Inno-Lia data of the present study. Thus, each notified Inno-Lia result was classified by each of these 10 algorithms as representing either an incident or older HIV-1 infection.

### Window periods, determination of incident infection ratio and statistics

The window periods of the selected 10 best algorithms, defined as the duration of HIV-1 infection after which, according to a given algorithm, all samples would be classified as representing an older infection, were taken from [18] and are listed in S1 Table.

The incident infection ratio (IIR), i.e., the proportion of incident among all newly diagnosed infections, was determined among those patients that had an Inno-Lia result, as described in the next two paragraphs. Annual numbers of incident infections were then obtained by multiplying the annual number of notifications with the IIR, thereby extrapolating missing Inno-Lia data.

For window-based calculations, the equation *IIR*_*W*_ = *n*_*tested incident*_/*n*_*tested*_ * 365/window was used, wherein *n*_*tested*_ equals the number of annual notifications [15]. The raw *IIR*_*W*_ thus obtained for each algorithm was furthermore adjusted for that algorithm’s pre-determined diagnostic specificity (*raw IIR*_*W*_ x %Specificity/100) [17], assuming that all assay parameters, i.e., window periods, sensitivity and specificity remained constant during the study.

Performance-based IIR (IIR_P_) was calculated based on the relationship n_tested incident_ = n_true- incident_ + n_false-incident_, wherein n_true-incident_ = n_tested_ × IIR_P_ × %Sensitivity/100 and n_false-incident_ = n_tested_ × (1 − IIR_P_) × (1 − %Specificity/100). Therefore, as published previously [14,17,18], IIR_P_ = (n_tested incident_ / n_tested_ + %Specificity/100 − 1) / (%Sensitivity/100 + %Specificity/100 − 1). Results below zero were set to zero. This model again assumes that the assay performance remains constant. Three different diagnostic sensitivities, S_1_, S_2_ and S_3_, were used; for details refer to [17]. In short, S_1_ averages the diagnostic sensitivities found in that study for each algorithm in the four quarters of the 12-months recent infection period. It corresponds to a model that assumes an even distribution of diagnosing incident infections over all four quarters. This model is probably incorrect, however, as many HIV-exposed individuals aware of their exposure will seek early clarification of their HIV status. Sensitivity S_2_ thus accounts for this bias by weighting the number of cases each quarter contributes to the total number of cases. Thus, the adjusted, weighted sensitivities S_2_ were calculated by multiplying the quarter sensitivities used for determination of S_1_ with the percentage of cases a quarter contributed to total cases and then averaging the products. Sensitivity S_3_ further adjusts for bias exerted by symptomatic patients, who are more likely to be diagnosed than asymptomatic individuals. For determination of S_3_, all cases judged incident because of reported signs or symptoms of ARS were excluded and only the notifications with a previous negative HIV test were considered for the calculation of diagnostic sensitivity. In comparison, sensitivity S_1_ is lowest, S_2_ is highest, and S_3_ is in-between; for details see the open access reference [17].

Correlations were assessed by Spearman’s rank correlation and linear regression analysis. Influence of variables on incident infection result was assessed by multivariate logistic regression. Calculations and statistical analyses were performed either in Microsoft’s Excel or StatView 5.0 for Macintosh (SAS Institute Inc., Cary, North Carolina, U.S.A.).

## Results

Between Jan 2008 and Dec 2013, a total of 3’851 individuals newly diagnosed with HIV infection were notified to the SFOPH (Table 1). Of this total of notifications, 3’699 (96.05%) contained Inno-Lia results, while the remaining 152 notifications (3.95%) didn’t. The proportion of notifications without Inno-Lia data diminished over time from 12.4% in 2008 to 6.5% in 2009, about 1.2% each in 2010 and 2011, and zero in both 2012 and 2013.

**Table 1:** Characteristics of the 3’851 HIV notifications received 2008–2013.

Annual notifications (n, %)		
** 2008**	768	19.94
** 2009**	658	17.09
** 2010**	606	15.74
** 2011**	564	14.65
** 2012**	648	16.83
** 2013**	607	15.76
**Sex (n, %)**		
** Male**	2820	73.2
** Female**	994	25.8
** unknown**	37	0.96
**Age, years (median, IQR)**	37	30─45
**Transmission mode (n, %)**		
** HET**	1428	37.08
** MSM (including 29 MSM/IDU)**	1438	37.34
** IDU**	103	2.68
** Transfusion** *	29	0.75
** Perinatal** **	21	0.55
** Unknown**	832	21.61
**HIV type (n, %)**		
** HIV-1**	3826	99.35
** HIV-2**	21	0.55
** Dual infection HIV-1 + HIV-2**	4	0.10
**With Inno-Lia results (n, %)**	3673	96.03

IQR, interquartile range;

* all infected abroad;

** predominantly infected abroad

Almost three-quarters of the notifications represented men. Transmission mode was dominated by HET and MSM, which represented about 37% each of the total. Intravenous drug use (IDU) was one order of magnitude less frequent (2.7%). Cases of transfusion-associated or perinatally acquired HIV infection totaling 1.3% were predominantly found in individuals infected abroad. The transmission mode was unknown (UKN) in 832 cases (21.6%). Totally, 3’826 patients were infected with HIV-1 (99.35%); 21 patients were infected with HIV-2, and 4 patients were dually infected with HIV-1 and HIV-2.

Assessment of incident infection in our study was restricted to HIV-1 infected persons that were at least 16 years old. Thus, the 21 perinatally acquired HIV-1 infections and the 21 HIV-2 infections were excluded, leaving 3’809 patients for the evaluation, of whom 3’636 (95.46%) presented with Inno-Lia data.

### Continuous decline of annual incident HIV-1 infections 2008─2013

The Inno-Lia data of these 3’636 adult notifications were subjected to evaluation by the 10 algorithms that distinguished best between incident and older HIV-1 infection, in order to decide for each notification whether it represented an incident (≤12 months) or older infection. For definition and diagnostic performance of these 10 algorithms, see S1 Table. Between a minimum of 413 (11.4%, Alg09) and a maximum of 685 notifications (18.8%, Alg04.1), on average 571 cases (15.7%), were ruled incident by these 10 algorithms (Table 2). On first glance, the number of infections ruled incident by the different algorithms decreased steadily between 2008 and 2013, but closer inspection showed that, in 2012, six algorithms ruled a higher number of infections incident than they did in 2011 (Algs 15.1, 15, 13, 07, 09, 04.1). The reason for this cannot be explained at present. On average, however, the number of cases ruled incident decreased from 2008 to 2011, plateaued in 2012, and decreased again in 2013.

**Table 2:** HIV-1 infections ruled incident by the 10 best-performing Inno-Lia algorithms.

Year	2008─2013	2008	2009	2010	2011	2012	2013
Total notifications	n = 3’636	n = 661	n = 599	n = 587	n = 549	n = 643	n = 597
	Number of notifications ruled incident						
**Alg15.1**	623	140	119	107	87	92	78
**Alg15**	644	141	122	110	93	97	81
**Alg11.2**	617	133	121	102	94	89	78
**Alg13**	560	126	106	95	81	82	70
**Alg07**	448	106	82	70	64	69	57
**Alg09**	413	100	75	66	58	62	52
**Alg11.1**	617	133	121	102	94	89	78
**Alg12.1**	627	136	122	105	96	89	79
**Alg04.1**	685	142	132	125	98	101	87
**Alg08.1**	480	110	91	76	73	69	61
**Mean**	571	127	109	96	84	84	72
**(%)**	(15.7)	(19.2)	(18.2)	(16.3)	(15.3)	(13.1)	(12.1)
**lower limit 95%CI**	514	117	97	84	75	76	65
**upper limit 95%CI**	629	136	121	108	93	92	79

Analysis restricted to notifications that contained Inno-Lia results.

**Fig 1:**
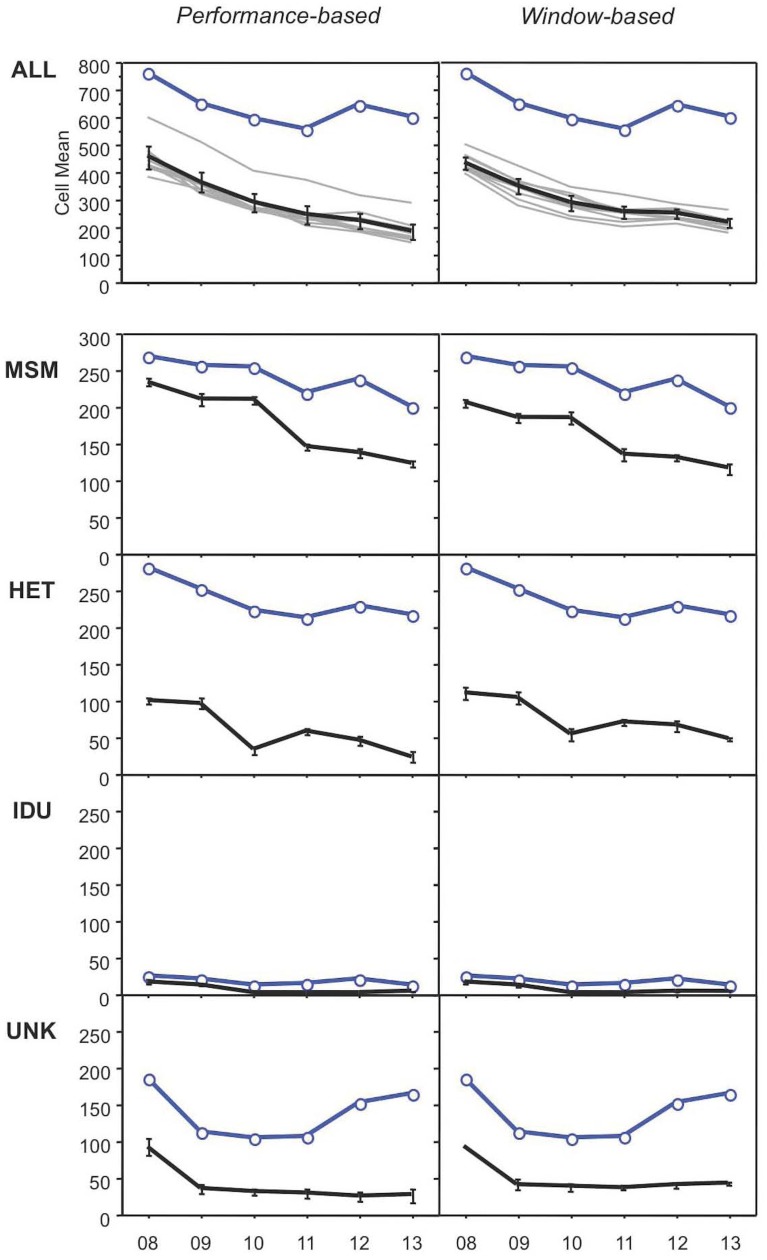
Number of HIV notifications and incident HIV infections over time, as obtained by performance-based and window-based incident infection estimates. Panels on top labeled “All” show the data for all patients, lower panels show the data per risk category (MSM, men who have sex with men; HET, heterosexual transmission; IDU, intravenous drug use; UNK, unknown transmission pathway). In all panels, the blue curve with the circle symbols denotes the annual number of HIV notifications, and the black curve without symbols shows the estimated number of incident infections (means and their 95% confidence intervals). The top panels also show the results obtained with the 10 individual algorithms (grey lines in the background).

The data of Table 2 were used to estimate annual incident infection ratios (IIR) in two ways, (i) based on the pre-determined diagnostic performance (IIR_P_), and (ii) based on the pre-determined window periods of the different algorithms (IIR_W_), as explained under Methods. Application of the respective IIR to the number of annual notifications (including those few that did not contain Inno-Lia data) then yielded the annual cases of incident infection. The calculations leading to these estimates are shown in detail in S2 Table. The estimates of annual incident infection for the 3’809 studied HIV-1 notifications, as well as their 95% confidence intervals (95%CI), are shown in Tables 3 and 4; they are also visualized in the top panels (labelled with “ALL”) of Fig 1.

**Table 3:** Incident HIV-1 infection estimates utilizing the performance-based method.

Year	2008─2013	2008	2009	2010	2011	2012	2013
Total notifications	n = 3’809	n = 763	n = 649	n = 597	n = 557	n = 643	n = 600
	Number of notifications estimated incident						
**Alg15.1**	1749	461	360	295	226	225	182
**Alg15**	1721	444	354	290	233	225	176
**Alg11.2**	1668	425	363	268	245	199	168
**Alg13**	1604	429	330	267	217	199	161
**Alg07**	1792	479	341	268	243	255	207
**Alg09**	1699	467	320	260	226	234	193
**Alg11.1**	1609	416	356	259	236	187	156
**Alg12.1**	1653	430	360	271	244	187	160
**Alg04.1**	1559	385	341	297	205	184	146
**Alg08.1**	2498	602	511	407	375	315	287
**Mean**	1755	454	364	288	245	221	184
**lower limit 95%CI**	1588	417	330	261	216	196	158
**upper limit 95%CI**	1923	490	397	316	274	246	209
**IIR**_**P**_ **(%)**	46.1	59.5	56.0	48.3	44.0	34.4	30.6

**Table 4:** Incident HIV-1 infection estimates utilizing the window-based method.

Year	2008─2013	2008	2009	2010	2011	2012	2013
Total notifications	n = 3’809	n = 763	n = 649	n = 597	n = 557	n = 643	n = 600
	Notifications estimated incident						
**Alg15.1**	1899	466	372	314	255	266	226
**Alg15**	1919	460	373	316	266	274	230
**Alg11.2**	1754	414	353	280	257	240	211
**Alg13**	1695	417	329	277	236	235	202
**Alg07**	1615	417	303	243	221	235	195
**Alg09**	1514	400	282	233	204	215	181
**Alg11.1**	1742	411	351	278	255	238	210
**Alg12.1**	1744	414	348	281	257	235	209
**Alg04.1**	1861	423	369	328	256	260	225
**Alg08.1**	2151	503	427	348	322	288	264
**Mean**	1790	432	351	290	253	249	215
**lower limit 95%CI**	1679	412	326	267	234	235	201
**upper limit 95%CI**	1900	453	376	312	272	262	229
**IIR**_**W**_ **(%)**	47.0	56.7	54.0	48.5	45.4	38.7	35.9

Both methods yielded comparable totals of incident infection estimates, amounting to 1’755 cases for the performance-based method (Table 3) and 1’790 cases for the window-based procedure (Table 4). Both methods indicated a continuous decrease of estimated annual cases of incident infections in dependence of time between 2008 and 2013, from a mean 454 cases to 184 cases (-59.5%) for the performance-based method and from a mean 432 to 215 cases (-50.2%) for the window-based method (Spearman’s correlation test, P = 0.0253 for both methods). Significant decreases by both estimation methods were also found for each of the 10 individual algorithms. The corresponding mean incident infection rates, IIR_P_ and IIR_W_, decreased to 51.0% and 63.3%, respectively, of their 2008 values (S2 Table). For 2012, when the notifications of new HIV diagnoses increased by 15% (from 557 cases in 2011 to 643 in 2012), neither method showed a rise in mean incident infections. Thus, the 15% increase in adult HIV-1 notifications of 2012 was caused entirely by a higher number of newly diagnosed older infections, while incident infections continued to decline in this year, though only minimally.

### Decline of annual incident infections for all major transmission modes

Assessment of incident HIV-1 infection for the different modes of transmission is shown in the lower panels of Fig 1; the underlying calculations for both performance- and window-based procedures are presented in S3 Table.

Among the total of 1’437 notifications attributed to MSM, as many as 1’060 notifications (73.8%) were estimated to represent incident infections based on IIR_P_, and 960 (66.8%) were estimated to represent incident infections based on IIR_W_. The annual estimates of 234 or respectively 206 incident cases for 2008 decreased to 122 (48% less than in 2008) or, respectively, 116 estimated cases for 2013 (44% less). These decreases in dependence of time were significant for both methods (Spearman’s, P = 0.0275). Both methods indicated a plateau of incident infections among MSM for 2009─2010, but neither method showed an increase in 2012, the year in which notifications among MSM had rebounded by 9% after three years of continuous decline (panels “MSM” of Fig 1; calculations in sections 3A and 3B of S3 Table).

Of the total of 1’417 notifications attributed to HET, 358 (25.2%) were estimated to represent incident infections based on IIR_P_ and 454 (32.1%) based on IIR_W_. Estimated annual cases of incident infections decreased from 100 or respectively 111 cases in 2008 to 24 (-76%) or 48 cases (-57%), respectively, in 2013. Both methods showed an over-proportional decrease of incident infections in 2010, followed by a transient increase in 2011 (panels “HET” of Fig 1; sections 1A and 1B of S3 Table). Spearman’s rank correlation test indicated a trend for decrease in dependence of time (p, 0.064 for both methods).

Of the 102 notifications attributed to IDU, 39 or respectively 43 cases were estimated to represent incident infections (38% or, respectively, 42%). The incident infection estimates of 17 (IIR_P_) or respectively 16 cases (IIR_W_) in 2008 decreased to 3 or respectively 4 cases in 2013, indicating a five- to four-fold reduction (panels “IDU” of Fig 1; sections 2A and 2B of S3 Table).

Finally, among the 826 notifications with unknown (UKN) mode of transmission, there were 239 (28.9%) or respectively 287 (34.7%) infections estimated incident. The annual figures decreased from 92 or respectively 93 in 2008 to 26 or respectively 42 in 2013, indicating reductions by 72% or, respectively, 55% (panels “UKN” of Fig 1; sections 4A and 4B of S3 Table).

Note that among all patients, the curves of annual notifications and annual incident infection estimates decreased in parallel between 2008 and 2011 (Fig 1, top panels). During this period, the differences between the annual numbers of notifications and estimated incident infections amounted to an average of 299 (95%CI 282─316) for IIR_P_ or, respectively, 305 (95%CI 278─332) for IIR_W_. After 2011, the two curves diverged, because the number of notifications increased while the incident infection estimates continued to decrease. From 2012 to 2013, both curves decreased again more or less in parallel, but now with a larger distance in-between; i.e., the difference increased to 420 for IIR_P_ (95%CI 384─456) and, respectively, 391 for IIR_W_ (95%CI 342─440). Thus, in 2012 and 2013, significantly higher numbers of older infections were diagnosed than in the preceding four years.

Parallel curves for notifications and incident infections between 2008 and 2011 were also observed for MSM and UKN, as well as for the few IDU (Fig 1, lower panels). Among HET, however, the two curves varied more, indicating that diagnoses of both incident and older infections varied considerably for consecutive years. Increases in notifications in 2012 were observed for MSM, HET, IDU and, particularly, UKN (here also for 2013), showing that in 2012 older infections were diagnosed more frequently in all transmission groups.

### MSM account for more than half of all incident infections

While total numbers of notifications were similar for MSM and HET (1437 and, respectively, 1417), MSM had a much higher proportion of incident infections. As a matter of fact, the majority of the incident infections were due to transmissions among MSM. According to the performance-based method, as many as 1060 (60.4%) of the mean estimated 1755 incident infections were attributable to MSM, while 358 (20.4%) were attributable to HET, 39 (2.2%) to IDU, and 239 (13.6%) to UKN (see Table 3 and S3 Table). Similarly, according to the window-based method (Table 4 and S3 Table), MSM accounted for 960 (53.6%) of the estimated total of 1790 incident infections, HET for 454 (25.4%), IDU for 43 (2.4%), and UKN for 287 (16.0%). As these are estimates and also partly because uncommon modes of transmission like transfusion-associated infection were not considered, the figures do not add up completely to the incident infection totals of Tables 3 and 4. A small percentage of these, 3.5% for performance-based and 2.6% for window-based, is not accounted for.

### Correction for selection bias

**Fig 2:**
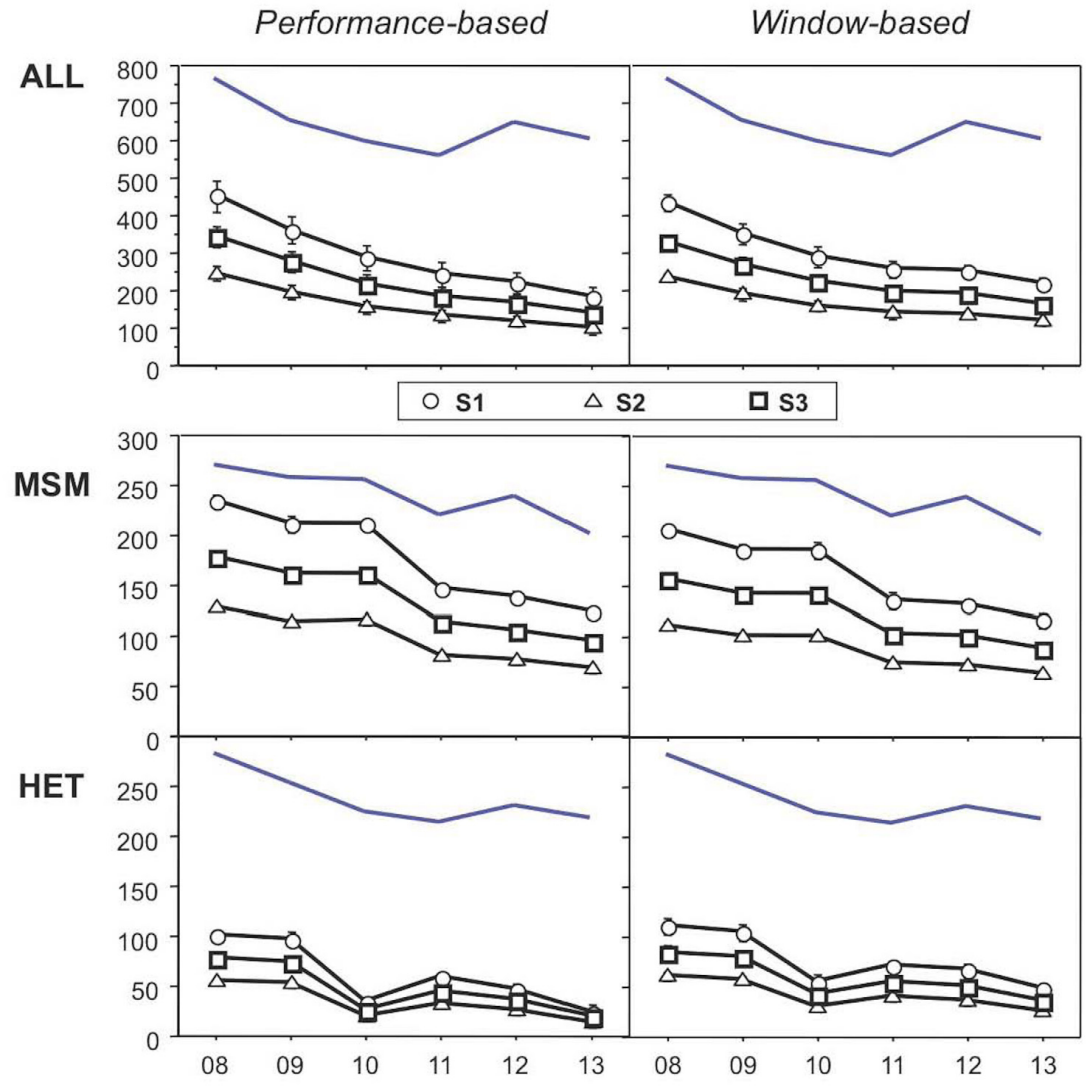
Incident infection estimates based on models adjusting for possible selection bias. S_1_, no adjustment; S_2_, model with adjustment for selection bias exerted by seeking early testing after a suspected exposure; S_3_, model with adjustment for seeking medical attention due to symptoms of acute HIV infection. Refer to Methods for further explanations. The blue curve without symbols on top in each panel shows the number of HIV notifications.

The incident infection estimates described above were obtained based on the assumption that diagnosis of incident HIV infection would be evenly distributed over all four quarters of the 12-months incident infection period. It is likely, however, that many HIV-exposed individuals seek early clarification of their HIV status, in particular if they experience symptoms of primary HIV infection (PHI). When using the performance-based method, such bias affects the diagnostic sensitivity of the algorithms [17]. Thus, by using sensitivity S_2_ (see Methods), adjustment is made for seeking early diagnosis after an experienced HIV exposure, and by using sensitivity S_3_ the bias exerted by symptoms of PHI is adjusted for. The results of these models are shown in Fig 2, and the underlying calculations are presented in S4 Table.

With adjustment for seeking early diagnosis (S_2_), annual incident infections were reduced to 54% of the numbers obtained with S_1_ and they dropped from 245 in 2008 to 99 in 2013. Analogous reductions were observed for the major transmission groups: for MSM, from 167 cases in 2008 to 66 in 2013; for HET, from 54 in 2008 to 13 in 2013. With the model adjusting for bias due to symptoms of PHI (S_3_), annual incident infections were reduced to 76% of the numbers obtained with S_1_, resulting in curves located in-between those of S_1_ and S_2_. Whatever the model, the decrease of annual incident infections from 2008 to 2013 remains the same: total incident infections dropped to 51% of their initial number.

Knowing now that adjustments for seeking early diagnosis (S_2_) or for symptoms of PHI (S_3_) reduce the numbers of incident infections to 54% and 76%, respectively, we applied these adjustments also to window-based incident infection estimates (Fig 2, panels on the right). This is justified, because a linear relationship between the diagnostic sensitivity of the algorithms and their window time has been demonstrated [18]. A proportional change in one of these two parameters is thus accompanied by an equal proportional change in the other.

## Discussion

Switzerland is one of the first countries with a comprehensive, country-wide system for monitoring incident HIV infections among newly diagnosed cases. The system is based on the results of the Inno-Lia which, since 2006, has been mandatory for confirmation of all newly diagnosed HIV infections and for differentiating between HIV-1 and HIV-2 [21]. Here we have evaluated the notification data collected by the SFOPH during the first six years since the system’s implementation in 2008 (Table 1). Based on Inno-Lia reaction patterns obtained prospectively during confirmation of a newly diagnosed HIV-1 infection, we determined the number of infections that were ruled incident by the 10 best-performing Inno-Lia incident infection algorithms (Table 2). We found that the average proportion of such incident pattern cases among the notifications decreased gradually from 19.2% in 2008 to 12.1% in 2013. Utilizing these empirically generated data and extrapolating them to all notifications, we estimated the proportion of incident infections in two ways, (i) based on the diagnostic performance of the algorithms (IIR_P_; Table 3), and (ii) based on the window periods of the algorithms (IIR_W_; Table 4). The two methods yielded comparable estimates of total incident infections amounting to 1755 and, respectively, 1790 cases, which corresponds to overall incident infection ratios of 46.1% and, respectively, 47.0%. The good agreement indicates that the diagnostic performance of the 10 selected algorithms on the one hand and their window periods on the other hand are, on average, correct.

Both methods showed a statistically significant, continuous overall reduction of newly diagnosed incident infections between 2008─2013 (Tables 3 and 4; Fig 1 top panels). Moreover, the reduction applied to all major transmission modes, again with similar curves for the two methods. Of note, incident infections continued to decrease in 2012 despite a 15% increase in HIV notifications observed in this year (Fig 1). Though numbers of notifications among MSM and HET were similar, MSM had a considerably higher proportion of incident infections. More than half of the estimated incident infections were attributed to MSM, 20─25% to HET and about 2% to IDU, while for the rest the transmission mode was unknown (Fig 1, lower panels).

### Limitations

That the observed decrease of HIV-1 infections ≤12 months duration among the newly notified cases is real, is supported by several lines of evidence. Statistically, the decrease was not only significant when the algorithms were used as a group, but also when used individually. However, when using these data for an estimation of HIV incidence in Switzerland, more elaborate statistics utilizing additional information like size and testing frequency of the different transmission groups will be indispensable for defining the true extent of the observed reductions and assessing the associated uncertainties [22].

The validity of our results depends on the assumption that assay performance, i.e. the sensitivity and specificity of the Inno-Lia and the algorithms used for result interpretation, remained constant during the study period. For some other TRI, notably the BED incidence assay, it has been shown, however, that the specificity depends on time since infection or, respectively, decreases in patients with very advanced disease [23,24]. Modelling studies in which the proportion of cases with advanced disease in an investigated population was varied have consequently shown that errors in incidence estimates based on the BED assay could vary by place, time and age-group, even if the proportion of truly incident infections did not change. Post-assay adjustment procedures like correcting for a specificity predetermined in a different population or established at a different time may thus not be valid in all situations [25]. We therefore have to demonstrate that the conditions under which our study was conducted did not change over time, or if they changed, that such changes could not explain the observed results.

With respect to assay performance, there is a possibility that the decline in incident infection numbers could be due to an inadvertent increase in sensitivity of the Inno-Lia over time, leading to bands of increasing intensity for a given plasma or serum sample. Similarly, bands of given intensity might over time be ruled to be of higher strength. Both types of variation would manifest themselves in our study as antibody reactions of increasing average intensity over time. We have checked such technical artifact by determining the average band intensity for all five HIV-1 antigens over time. While there was some variation among both recent and older infections, there was no trend for increase that could be correlated with, and explain, the observed continuous reduction of incident infections (S2 File).

Another possible source of error relates to the assumption that the diagnostic specificity of the Inno-Lia algorithms remained constant during the study. As HIV-specific antibody titers decrease with disease progression [26–30], TRI may become false-recent. Thus, if the proportion of patients with advanced disease in an investigated population increases over time, this may result in an apparent increase of incident infections and vice-verse. As we observed a decrease, we investigated whether this could be due to a reduction of patients with advanced disease over time. This was not the case: the proportion of notifications with AIDS at diagnosis increased from 10.3% in 2008 to 15.6% in 2010, and then decreased again to 9.3% by 2013. Previous work has shown furthermore that CDC stage C does not impair the specificity of the Inno-Lia algorithms [16]. In contrast to TRIs like the BED incidence assay or tests based on antibody affinity, which are solely based on signal/cutoff ratios, the Inno-Lia algorithms in addition define band patterns that differ between early and late infection. In early infection, reaction to p24 and p17 is generally stronger than to p31, because antibodies to p31 are the last to appear [18,31,32]. In advanced infection, however, antibody reaction to p31 and the envelope proteins is preserved, while that to p17 and p24 is diminished, due to an early down-regulation in patients with disease progression [26–30]. Most of the Inno-Lia algorithms used in this study specify that reaction to p17 and/or p24 be higher, or at least not lower, than that to p31 (see S1 Table). This largely preserves the specificity in advanced infection.

It has also been reported that a low HIV viral load, be it as a consequence of ART or as seen in elite virus controllers, is associated with a delayed antibody response and lower titers of HIV-specific antibody titers, thus resulting in false recent TRI; this was also found for the Inno-Lia algorithms [16,23,24,33–35]. However, since we perform the Inno-Lia at the time of HIV diagnosis, ART-induced false-incident results should be absent. Still, a sizeable and over time increasing presence of elite controllers in our study could have such an effect. This was not the case, however, as the proportion of patients with viral load ≤50 copies/mL was only 1.14%, though rising from 0.47% in 2008 to 2.3% in 2013. Of note, however, the rise did not occur among infections ruled incident, but among those ruled older. Thus, this mechanism did not contribute to the observed 50% reduction of incident infections.

It has furthermore been suggested that individuals with a low HIV viral load are enriched over time in a population, due to their higher probability to survive [25,33]. While this may apply to countries where good medical care with access to ART is unavailable to the majority of the inhabitants, such selection does not operate in a country like Switzerland that has an excellent health care system accessible for all its inhabitants. Furthermore, it is unconceivable that such selection could take place within the six years of the study. As a matter of fact, as stated above, the proportion of cases with a viral load ≤50 was only 1.14%. Actually, 26 of the 31 such cases were ruled older and, among these, the proportion increased from 0.6% in 2008 to 2.4% in 2013. Among cases ruled recent, there was never more than a single case per year. Thus, such selection did not contribute to the observed decrease in incident infections.

Regarding age, it has been recognized that older population groups have a higher chance of being in advanced stage of infection compared to young adults whose risk of getting infected started only when reaching adolescence. With the BED incidence assay, older HIV positive populations therefore also have a higher proportion of patients in advanced disease, thus resulting in more false-recent TRI results [25]. In order to investigate this possibility, we assessed the age of our patients over time and found no difference. Mean age amounted to 37.8 years in 2008, increased to 39.2 years in 2011, and decreased again to 38.4 years in 2013.

In order to further assess the influence of these factors when present in combination we performed multivariate logistic regression. We used a model in which transmission risk, diagnosis of AIDS in the year of diagnosis, sex, year of diagnosis, viral load ≤50 copies/mL and age were the independents and incident infection result by the top-ranking Alg15.1 was the dependent. These data were available for 2700 cases, i.e. about 75% of the notifications with Inno-Lia results. Two variables promoted an incident infection result: MSM compared to HET (p<0.0001; odds ratio (OR), 1.963; 95% CI, 1.511–2.552), and being AIDS-free (p<0.0001; OR, 3.739; 95% CI, 2.437–5.738). Two variables were associated with a decreased risk of an incident infection result, namely female sex (p = 0.0015; OR, 0.606; 95% CI, 0.445–0.852) and increasing year of diagnosis (p<0.0001; OR, 0.883; 95% CI, 0.833–0.936). Low viral load and age carried no significance (p>0.7). Similar results were obtained for the other nine algorithms. Adding CD4^+^ counts to this model reduced the available number of cases to 850. Having a *high* CD4^+^ T cell count clearly promoted a result of incident infection (p = 0.0002; OR per additional 100 CD4^+^ cells, 1.125; 95% CI, 1.058–1.195). Being an MSM and being AIDS-free at diagnosis retained significance in this model, while all other variables were non-significant. Thus, in contrast to the findings with the BED incidence EIA, but in agreement with our earlier findings [16], the specificity of the Inno-Lia algorithms was not impaired by age, viral load, or advanced stage of disease. We thus conclude that the limitations that apply to the use of the BED incidence EIA as shown by [25] do not apply to our study and that the assay performance characteristics, the diagnostic specificity and sensitivity, remained constant during the entire study period.

A weakness of most incident infection estimates is the fact that they are usually based on the probably incorrect assumption that incident HIV infection will be diagnosed at a constant frequency during the 12 months of the incident infection period. Indeed, it has been demonstrated that illnesses associated with seroconversion and other factors promote diagnosis within the initial quarter of this first year after infection, and methods for correcting such bias have been presented by others [36]. In our study, we have also addressed this point and have used two models that adjust for such selection bias (Fig 2). Although it is unknown which of the models is the best one, they indicate the effects exerted by such biases. Independently of the choice of model, the three models show that the proportion of incident HIV infection was reduced by half during the study.

As with other tests, the reliability of Inno-Lia based incident infection assessment depends on the prerequisite that all factors other than the investigated variable remain unchanged during the study. The Swiss dual notification procedure with both a laboratory and a physician’s notification (see Methods) has been in operation for more than 20 years now and also provides for effective recognition and exclusion of repeat notifications. As stated under Results, the proportion of notifications with Inno-Lia result increased from 87.6% in 2008 to 100% in 20012 and 2013. It is difficult to see, however, how this change should have affected the proportion of incident infection patterns among the notifications. The completeness of laboratory reporting has also improved during the study; cases with a physician’s notification alone have entirely disappeared in recent years. But again, it is difficult to see how this change could have biased the results. We thus assume that the observed decrease in the proportion of incident HIV infections was not due to changes in the reporting system.

On the other hand, changes in testing behavior might also have influenced the results over time. For example, even if the number of newly acquired infections didn’t change, the incident infection ratio and, thus, the number of patients ruled to have incident infection, would decrease if a sufficiently large proportion of the newly infected individuals chose to get tested just a few months later than in previous years. However, in view of the fact that early HIV testing has been recommended vigorously in recent years and of growing evidence that early initiation of ART is beneficial for the long-term outcome of HIV-1 infection (evidence reviewed in [37]), such an effect is unlikely to have impacted our study.

A distinct increase in diagnoses of syphilis and other sexually transmitted diseases was observed among MSM between 2009 and 2013 in Switzerland, indicating that, as in other countries, risk-taking behavior increased in this group [2]. It is thus surprising that during the same time the number of observed incident infections should have decreased also among MSM, and the question arises whether the observed decrease could be due to selection bias. Could MSM with recent HIV infection be underrepresented in the present study? Data collected in voluntary counseling and testing (VCT) centers in Switzerland, particularly the so-called ‘Checkpoints’ targeted to the special needs of MSM, indicate that this is probably not the case. HIV testing among MSM in such VCT centers increased about three-fold between 2009 and 2013, while testing among heterosexual men and women increased only little. In addition, based on information contained in the physicians’ supplemental notifications (see Methods), only 8% of the MSM, but 14% of the other transmission risk groups were considered to be late presenters. Together these data suggest that HIV testing among MSM was more frequent than among the other groups [2]. Thus, if there was bias at all, it was probably in favor of MSM with recent HIV infection. Provided that the situation in VCT centers is representative of the total, HIV incidence in MSM across Switzerland may thus have decreased even more than suggested here. A clear assessment of HIV incidence is not possible, however, as numbers of HIV tests performed in the country are unknown.

### Causes for the decrease in newly diagnosed incident HIV infections

The causes of the decline of newly diagnosed incident HIV infections in Switzerland remain unknown, but in theory may include any of the following: improved preventive behavior in the population particularly at risk for HIV infection, earlier diagnosis of HIV infection, earlier start of antiretroviral treatment (ART) resulting in fewer untreated patients and, therefore, a lower risk of secondary transmission. The promotion of HIV testing among individuals at risk has been a central goal of the national HIV prevention campaign during the past decade, but statistics for the total number of HIV tests conducted each year in our country are lacking. That the number of newly diagnosed HIV-1 infections of more than 12 months duration increased significantly in 2012─2013 (Fig 1, top) may indicate that pre-existing infections were diagnosed more efficiently, possibly in response to an extension of the national HIV prevention program to other sexually transmitted infections (STI) implemented in 2011 [38]. The effort to increase public awareness to STI may have led to a higher detection of pre-existing HIV infections in individuals seeking medical assistance due to signs and symptoms of STI.

International treatment guidelines have recommended an ever earlier start of antiretroviral treatment (ART). Guidelines implemented in the U.S. in 2012 recommend that all HIV infected individuals receive ART while, in Europe, the use of ART should at least be considered and actively discussed with the HIV-positive person [36,39]. As a consequence, the community viral load and the number of individuals with an HIV load sufficiently high for virus transmission are expected to decrease more and more, leading to fewer and fewer transmissions [40–42]. The statistics of the Swiss HIV Cohort Study (SHCS), in which significant proportions of the HIV-infected individuals in Switzerland participate, namely 53% of the patients with an HIV notification and 69% of the patients with an AIDS notification to the SFOPH, show that the median CD4+ T-cell count at the initiation of ART in treatment-naïve patients has constantly increased in recent years, particularly among MSM. Similarly, the proportion of non-treated patients in the SHCS has continuously decreased from 20% in 2007 to 5.6% in July 2012 [43]. These data are compatible with, but not proof of, the notion that increased antiretroviral treatment of the HIV infected community may indeed have contributed to the observed decrease of incident infections. A decrease in the community viral load would best explain the changes; a study on that in the SHCS is in progress, but results are not yet available.

### Strengths and weaknesses of Inno-Lia based incident infection estimation

One major advantage of the Inno-Lia based estimation of incident HIV infection is the availability of a whole panel of algorithms, each with its own diagnostic sensitivity, specificity and window length (S1 Table). In contrast to other TRIs, where there is just one test or algorithm, Inno-Lia thus provides a whole panel of tools for assessing each specimen. This has distinctly more power to identify true changes in the proportion of incident infections than any other single serologic TRI. As can be seen in Fig 1 (top panels), there is considerable variation in the incident infection estimates based on individual algorithms. We have pointed out previously that it would be impossible to select a “best curve”, and the reliability derives from the combination of different algorithms [17]. The low and changing specificity of the BED incidence assay [25,44] has prompted others to resort to additional criteria for assessment of incident HIV infections. A multi-assay algorithm for HIV incidence that includes the BED incidence EIA, an antibody avidity assay, HIV load, and CD4^+^ T-cell count has been shown to have excellent specificity [45]. This approach has also been used successfully in order to determine HIV incidence in cohort studies in which the needed additional information is easily available [46]. At the time of HIV diagnosis, such information is frequently not available, however, and the advantage of our model consists of the fact that such information is not needed and that no additional test has to be performed. Thus, similar to our approach, a bead based multiplex assay that measures both the levels and the avidity of antibodies to various HIV-1 antigens and detects recent HIV-1 infections with high sensitivity, specificity and robustness has been developed and evaluated recently [47,48].

Regarding the Inno-Lia, the use of an HIV confirmation assay for incident infection surveillance is a further advantage, because data were available from virtually all newly diagnosed patients. Once the system was well established, i.e., from the third year onward, 98% or more of the notifications contained Inno-Lia data. Therefore, the incident infection estimates are truly representative. In comparison, the French monitoring system for incident HIV infection, which combines a mandatory notification of all newly diagnosed cases with an enzyme immunoassay for recent infection performed on dried serum spot samples sent to a central laboratory, had an overall coverage of 76.5% between 2003 and 2008 [11]. The British monitoring system, which combines a mandatory case notification with an avidity assay performed in a central laboratory, had an avidity test coverage which increased from 24% in 2009 to 52% in 2011 and averaged 38.4% [12].

Inno-Lia based incident infection surveillance does not require additional tests, nor is shipping of samples to a central lab required. Whether one uses a single algorithm or a combination of different ones has no effect on costs, as these population-based evaluations, once set up, can be performed in an automated way, e.g. by pasting the Inno-Lia dataset into a simple, pre-formed Microsoft Excel-based evaluation table.

We demonstrated earlier that the result of the Inno-Lia algorithms in ART-naïve patients is not affected by the severity of the immunodeficiency [16]. Therefore, Inno-Lia based surveillance of incident infection does not require prior exclusion of the patients who are in an advanced stage of disease. We showed in the same study that the clade of HIV-1 does not influence the incidence result. Technically, the method should thus also be feasible for countries that have an HIV-1 clade distribution different from that of Switzerland, where subtype B dominates the newly diagnosed infections with about 60% (analysis based on the sequences of 2670 new patients entered into the national HIV resistance database from 2009–2012). Of course, such a transfer would need extensive validation prior to implementation.

The Inno-Lia is a relatively costly test that will be unaffordable for many low-income settings. However, for countries already using this test, its further utilization for nationwide assessment of incident infection can provide an additional public health benefit that is obtained at virtually no additional costs. In the Swiss testing system, Inno-Lia is restricted to patients with a reactive result in two, preferentially different, 4^th^ generation screening tests, i.e. two independent tests of equally high diagnostic specificity [49,50]. Thus, only few Inno-Lia tests are performed unnecessarily due to false-positive screening result. At a specificity of 99% for each of the two screening tests (the CE-marking rules actually request a specificity of 99.5% or higher [51]), the combined specificity is 99.99%. Out of 100’000 tested uninfected individuals only 10 would have undergone Inno-Lia testing in addition to those truly infected. Based on the 3’851 HIV-positive individuals of this study, and assuming that maximally 3 million HIV screenings were conducted in Switzerland during the six years, it is expected that 3’851 + 300 = 4’151 Inno-Lia tests would have been conducted in total. At the current compensation of CHF 132 per test, the total costs for the Inno-Lia testing amounted to approximately CHF 548’000, or CHF 92’000 per year. This is less than 1% of the costs of HIV screening which, at CHF 20 per test, would amount to 10 Million CHF per year. Based on HIV-prevalence and kit prices in other countries (the latter most likely being lower than in Switzerland), costs are likely to differ for other settings.

In conclusion, our Inno-Lia based surveillance system shows a continuous overall reduction of incident HIV-1 infections by 50% in Switzerland from 2008 to 2013, even for 2012 when notifications increased by 15%. The decline was true for all major transmission modes, MSM, HET and IDU. As the Inno-Lia algorithms perform equally well for the various HIV-1 subtypes, the method should be technically feasible also for countries that have an HIV-1 subtype distribution different from that of Switzerland. The Inno-Lia based assessment of incident HIV infection ratios can be performed without a need for clinical information other than that the patients are treatment-naïve, a requirement always met when newly diagnosing HIV infection in an individual patient. Cost-wise, the system is particularly attractive for countries which already use the Inno-Lia for confirmation of HIV infection and differentiation between HIV-1 and HIV-2. For these, the Inno-Lia based incident infection assessment represents a free, additional public health benefit of the use of this relatively costly test.

## Supporting Information

S1 Dataset Complete Excel dataset used for this study. (XLS)

S1 Fig Principle of Inno-Lia based assessment of incident HIV-1 infection exemplified on two Inno-Lia strips, one representing an incident infection and the other representing an older infection. (PDF)

S1 File Description of the Swiss HIV Testing Concept 2013 and of the dedicated Excel-based macro-sheet for country-wide standardized HIV confirmation and case notification to the SFOPH. (The macro-sheet and assistance for necessary adaptations are available on request from JS).
(PDF)

S2 File Assessment of assay sensitivity over time. (PDF)

S1 Table Table listing the 10 best-performing Inno-Lia algorithms used in this study. (PDF)

S2 Table Detailed calculations for the results of Tables 3 and 4 and the top panel of Fig 1. (PDF)

S3 Table Detailed calculations for the results presented in the lower panels of Fig 1. (PDF)

S4 Table Detailed calculations for the results presented in Fig 2. (PDF)
