# Supplementary material for: Decreasing Proportion of Recent Infections among Newly Diagnosed HIV-1 Cases in Switzerland, 2008 to 2013 Based on Line-Immunoassay-Based Algorithms
Source: PLoS One. 2015 Jul 31;10(7):e0131828. doi: 10.1371/journal.pone.0131828 (PMC4521810; doi:10.1371/journal.pone.0131828)

**S1 Fig**

**Principle of Inno-Lia based assessment of incident HIV-1 infection.**

The Inno-Lia incident infection algorithms are based on the intensity of a patient's antibody reactions with the five HIV-1 antigen bands on each test strip, gp120, gp41, p31, p24, p17.

The reaction intensity is obtained by comparing, according to the manufacturer's instruction, each antibody reaction band with the three intensity control bands located at the top of each strip. The test strip on the left shows an early infection pattern with antibodies to gp41 at intensity of about 1 and to p24 at intensity 0.5. The strip on the right shows a more advanced stage with reaction to all antigens of HIV-1; the intensities are 0.5 for anti-p17, 3 for anti-gp41, and 2 each for anti-gp120, anti-p31 and anti-p24.

The reaction patterns of each patient are subjected to interpretation by each of the 10 algorithms listed in **S1 Table**. Each of these algorithms will rule that the strip on the left represents an incident infection and that the strip on the right represents an older infection. For less clear results the interpretations may differ.

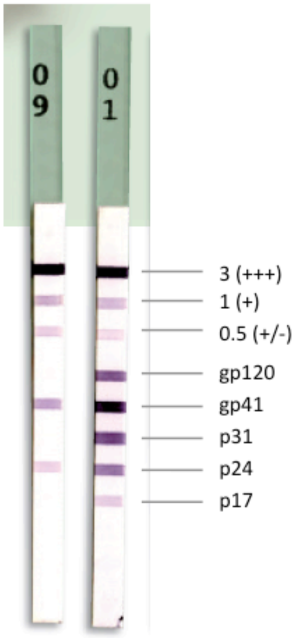

Supplement: S1 Fig — (PDF) [file pone.0131828.s002.pdf]
