# Supplementary material for: Decreasing Proportion of Recent Infections among Newly Diagnosed HIV-1 Cases in Switzerland, 2008 to 2013 Based on Line-Immunoassay-Based Algorithms
Source: PLoS One. 2015 Jul 31;10(7):e0131828. doi: 10.1371/journal.pone.0131828 (PMC4521810; doi:10.1371/journal.pone.0131828)
Supplement: S1 File — (The macro-sheet and assistance for necessary adaptations are available on request from JS). (PDF) [file pone.0131828.s003.pdf]

Universität Zürich[UZH]

Institute of Medical Virology
Swiss National Center for Retroviruses
Winterthurerstrasse 190
CH-8057 Zürich

**HIV Testing Concept 2013 of the Swiss Federal Office of Public Health**

# An experimental Excel®-based tool for country-wide standardized HIV confirmation and case notification

HIV confirmation in Switzerland is guided by the **HIV Testing Concept** set as the diagnostic standard by the Swiss Federal Office of Public Health (SFOPH). In order to facilitate a rapid, country-wide and standardized implementation of the Concept's comprehensive guidelines into the daily diagnostic routine of the **11 HIV Notification Labs**, the Swiss National Center for Retroviruses (SNCR) has developed an experimental electronic tool (hence called "the Tool") which guides through all steps of the confirmation process. With each new result entered, the Tool summarizes what has been achieved with respect to the four principal questions of HIV diagnosis (Table 1) and what has still to be done. The Tool also generates an Overall Case Interpretation which is sent to the patient's physician as a brief information about the diagnostic state regarding the Concept's requirements. The Tool also generates an anonymized electronic case notification to the SFOPH.

The Tool is based on the Microsoft Excel® program, rendering it instantly useable to all 11 Notification Labs without further software expenditures. A first prototype was developed in 2007 and evaluated in a 4-months internal study. During this time, the Notification Labs entered the results of all newly diagnosed HIV infections into the Tool and examined whether it worked properly and yielded adequate Overall Case Interpretations. Malfunctions were prospectively reported to the SNCR. Based on these, the SNCR continuously improved the prototype until it was deemed fit for the next step of the evaluation. From September 2007 onwards, the Tool was used with all of its functions. The Overall Case Interpretation and Case Notification it generated were sent to the treating physician and the SFOPH, respectively, while still keeping a watchful eye on possible malfunctions and eliminating them in successive new versions of the Tool. At the end of 2008, the evaluation was completed and the SFOPH ruled that use of the Tool was to be continued as the standard means for HIV confirmation and case notification.

Since 2008, the SFOPH is in possession of complete Tool-generated anonymized data on all newly diagnosed cases of HIV infection. These data provide an important basis for the national HIV surveillance. In particular, the patients' antibody reaction patterns in the HIV line immunoassay have been used routinely since 2008 for estimating the proportion of fresh infections (≤12 months) among the newly diagnosed HIV infections and to monitor changes of this proportion over time among the different transmission risk groups [1,2,3].

---

The benefits of the Tool as a means to implement the Swiss HIV Testing Concept are as follows:

**Standardization of HIV confirmation** in a decentralized federalistic system. In Switzerland, HIV confirmations are performed in one of the 11 HIV regional Notification Labs or the SNCR. These labs are located in three different language areas. Some are associated with universities or other public organizations, while others are private, commercial labs. The system works equally well in all settings. With exception of the Inno-Lia™ HIV I/II Score assay, which has to be performed by all, the labs are free in their selection of tests, provided that they are in agreement with the specifications of the HIV Test Concept. For example, for screening, the labs may use any CE-marked 4$^{th}$ Generation test according to their individual needs and preferences.

**Sustained improvement of diagnostic quality.** The fact that the Tool-generated diagnostic data are sent to and archived at the SFOPH, where they can be examined at any time, contributes to an improved quality of the work in the labs. This effect may be more sustained than periodical audits by an accreditation agency.

**Improved diagnostic safety.** That all new HIV diagnoses are examined by an independent expert

at the SNCR increases their safety. No false HIV diagnoses anymore!

**Country-wide anonymized diagnostic data for scientific evaluation.** The SFOPH has the possibility to scientifically evaluate the collected data with respect to the diagnostic performance of the various tests, costs etc.

**A cost-limiting effect.** Employing the Tool makes its users aware of the fact that in most instances very few tests are needed to meet the requirements of the HIV Testing Concept. Usually, a reactive HIV screening test on the first sample and a positive line-immunoassay, a viral load test and a genetic resistance test on the second sample is all that is needed to fulfill the Concept's requirements.

**Estimation of the rate of incident HIV infections.** The annual datasets contain near-complete information on Inno-Lia reactivity (>99% of all notified cases), thus permitting accurate estimation of the annual proportion of incident infections in the country.

**Country-wide information and education of physicians.** The Tool-generated Overall Case Interpretation sent to the treating physicians makes them aware of the diagnostic requirements of the HIV Testing Concept. It remains the physician's responsibility to ensure that all demands of the Concept are met.

## Features of the Tool

The Tool consists of the following sections:

### 1. Recording information required for case notification to the SFOPH

a. the laboratory identification number
b. date of birth
c. sex
d. first-name code according to SFOPH requirements
e. sample date
f. canton of residence
g. name and address of the treating physician
h. name of the Notification Lab
i. test setting (VCT Checkpoint, anonymous screening, blood donor screening, other)

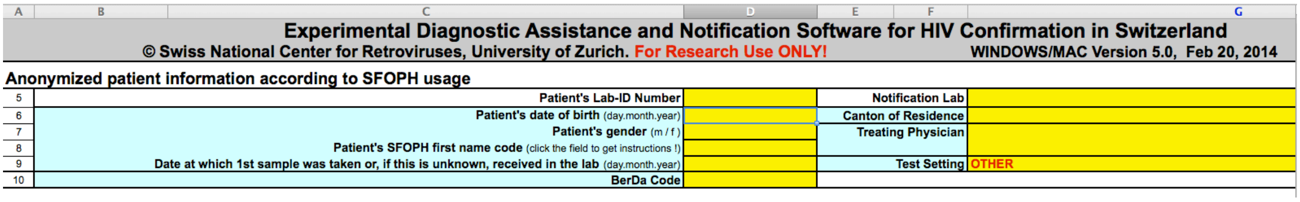


The yellow fields are open to data entry, all others are blocked.

### 2. Recording pre-existing result information generated in other labs

If available, the Tool utilizes result information that was generated by accredited medical-diagnostic laboratories or physician's offices and was communicated to the Notification Lab. Such information may include the following:

a. Result of a rapid 3rd generation HIV screening test (antibody only)
b. Result of a rapid 4th generation HIV screening test (antibody + p24 antigen)
c. Result of a 4th generation HIV screening test conducted in the laboratory

The tool also records whether these tests were performed on the same sample as that received by the Notification Lab or from an earlier one. This is important for the mix-up exclusion criterion of the HIV Testing Concept (see Table 1, 1st question).

| Outside results (from clinical labs, doctors' offices, VCT centers) | | RESULT |
|---|---|---|
| 12 | HIV-1/2 Rapid *Antibody Only* Assay Result (pos=1 ; ind=0.5; neg=0) | |
| 13 | HIV-1/2 Rapid *Ab+Ag Combo* Assay (pos=1 ; ind=0.5; neg=0) | |
| 14 | 4th Generation HIV-1/2 Ab/Ag EIA (pos=1 ; ind=0.5; neg=0) | |
| 15 | Were the above test(s) done on a sample different from that received by the CL? (YES/NO/UNKNOWN) | |

Results are entered by numbers: 1, pos; 0, neg; 0.5, indeterminate. More complicated entries are made by means of a pull-down menu (YES/NO/UNKNOWN).

## 3. Recording serology results generated by the Notification Lab on the first sample it received for testing

This section serves to record the serological results generated by the Notification Lab with the first specimen it received for HIV confirmation. The results that can be entered into this section include the following:

a. 4th generation HIV-1/2/O antibody + antigen assay, Test A
b. 4th generation HIV-1/2/O antibody + antigen assay, Test B
c. 3rd generation HIV-1/2/O antibody only assay
d. HIV-1 p24 antigen assay, screening
e. HIV-1 p24 antigen assay, neutralization
f. Inno-Lia™ HIV I/II Score assay, antibody signal intensity on all 7 bands representing HIV-1 and/or HIV-2 antigens.

| Tests done on the 1st sample that was received by the Notification Lab | | | RESULT |
|---|---|---|---|
| 19 | 4th Generation HIV-1/2 Ab/Ag Combination EIA; Test A (pos=1 ; ind=0.5; neg=0) | | |
| 20 | (Signal/Cutoff Ratio) | | |
| 21 | | | |
| 22 | 4th Generation HIV-1/2 Ab/Ag Combination EIA; Test B (pos=1 ; ind=0.5; neg=0) | | |
| 23 | (Signal/Cutoff Ratio) | | |
| 24 | | | |
| 25 | 3rd Generation HIV-1/2 *Antibody-ONLY* EIA (pos=1 ; neg=0) | | |
| 26 | (Signal/Cutoff Ratio) | | |
| 27 | | | |
| 28 | HIV p24 Antigen EIA Screening (pos=1 ; neg=0) | | |
| 29 | (Signal/Cutoff Ratio) | | |
| 30 | | | |
| 31 | HIV p24 Antigen Neutralization (pos=1 ; neg=0) | | |
| 32 | | | |
| 33 | *The subsequent Inno-Lia data originate from:* | | *this lab* |
| 34 | Inno-Lia HIV-1/2 | sgp120 (HIV-1) | |
| 35 | Score, 1st Sample | gp41 (HIV-1) | |
| 36 | (enter all reactions | p31 | |
| 37 | including those negative | p24 | |
| 38 | with a score [0, .5, 1, 2, 3 or 4]) | p17 | |
| 39 | | sgp105 (HIV-2) | |
| 40 | | gp36 (HIV-2) | |

In addition to the qualitative test result (pos, neg, ind), signal/cutoff ratios may be recorded. The ratios have no impact on overall result evaluation however. For Inno-Lia, antibody intensity is scored by grades 0 (no reaction), 0.5 (+/-), 1, 2, 3, 4 according to manufacturer's instructions and using the semiquantitative standards positioned on each test strip.

## 4. Results of nucleic acid tests (NAT)

NAT can be done on the first or a later sample received by the Notification Lab. If the first sample is a plasma, molecular tests such as viral load assay or resistance testing can be done already on that first sample. Otherwise, a second sample (EDTA-blood) has to be requested for these tests.

a. Roche COBAS Taqman HIV-1 RNA Quantitative
b. Abbott real-time HIV-1 RNA Quantitative
c. HIV-1 DNA qualitative

These first three tests are commercial assays used by both the Notification Labs and the SNCR. Most Notification Labs perform the Roche assay, but some also perform the Abbott assay or, occasionally, PCR for HIV-1 DNA.

| | **Commercial nucleic acid tests** (mark whether done on 1st or 2nd sample!) | RESULT | Sample |
|---|---|---|---|
| 52 | **ROCHE COBAS Taqman HIV-1 Monitor RNA Quantitative** (copies/mL; undetectable = 0) | | |
| 53 | | | |
| 54 | **Abbott real-time HIV-1 RNA Quantitative** (copies/mL; undetectable = 0) | | |
| 55 | **HIV-1 DNA qualitative** (pos=1; neg=0) | | |

The Tool requires that the sample (1st, 2nd or 3rd) on which these tests are performed, is indicated. This is important for the criterion "at least two different samples have a positive result"; see Table 1).

A second group of tests (d to i) is offered only by the SNCR and is used for special, complicated cases (consult the **HIV Testing Concept**). This second group includes the following tests:

d. PERT assay quantitative (a test for quantification of HIV-1 or HIV-2 based on the reverse transcriptase activity contained in the viral particles)
e. HIV-2 PCR, qualitative
f. HIV-1 Group-O specific real-time PCR for DNA or RNA, qualitative
g. HIV-1 Group-M specific real-time PCR for DNA or RNA, qualitative
h. HIV-1 DNA; ultrasensitive, high-input (MEGA) real-time PCR, qualitative
i. HIV-1 DNA; ultrasensitive, high-input (MEGA) real-time PCR, qualitative

| | **Nucleic acid test results** (may be obtained from NZR; mark whether on 1st, 2nd, or 3rd sample) | | |
|---|---|---|---|
| 57 | **PERT Assay Quantitative** (converted into HIV-1 copies/mL; undetectable = 0) | | |
| 58 | | | |
| 59 | **HIV-2 SPECIFIC DNA Qualitative** (pos=1; neg=0) | | |
| 60 | **HIV-1 GROUP O-SPECIFIC DNA or RNA; Real-Time PCR** (pos=1; neg=0) | | |
| 61 | **HIV-1 GROUP M-SPECIFIC DNA or RNA; Real-Time PCR** (pos=1; neg=0) | | |
| 62 | **HIV-1 DNA, High Input (MEGA) Real-Time PCR, Qualitative** (pos=1; neg=0) | | |
| 63 | **HIV-2 DNA, High Input (MEGA) Real-Time PCR, Qualitative** (pos=1; neg=0) | | |

## 5. Recording serology results generated by the Notification Lab on the 2nd sample it received for testing

Although most serologic testing is done in the first sample, it is occasionally useful to perform some serological tests on the second sample received for HIV confirmation. For example, if due to primary HIV infection the Inno-Lia in the first specimen was still negative, a repeat test in a second sample taken a few weeks later may indicate the HIV type; and the increase in antibody reaction will confirm the acute infection. Thus, the same tests results as for the first sample may be entered:

a. 4th generation HIV-1/2/O antibody + antigen assay, Test A
b. 4th generation HIV-1/2/O antibody + antigen assay, Test B
c. 3rd generation HIV-1/2/O antibody only assay
d. HIV-1 p24 antigen assay, screening
e. HIV-1 p24 antigen assay, neutralization
f. Inno-Lia™ HIV I/II Score assay, antibody signal intensity on all 7 bands representing HIV-1 and/or HIV-2 antigens. Change in band intensity compared to the Inno-Lia result with the first specimen (if performed) will be calculated automatically and considered for overall case interpretation.

| | **Tests done on the 2nd or a 3rd sample received in the Confirmatory Lab** | | | (Signal/Cutoff Ratio) |
|---|---|---|---|---|
| 67 | | **4th Generation HIV-1/2 Ab/Ag Combination EIA; Test A** (pos=1 ; ind=0.5; neg=0) | | |
| 68 | | **4th Generation HIV-1/2 Ab/Ag Combination EIA; Test B** (pos=1 ; ind=0.5; neg=0) | | |
| 69 | | | | |
| 70 | | **3rd Generation HIV-1/2 *Antibody-ONLY* EIA** (pos=1 ; neg=0) | | |
| 71 | | | | |
| 72 | | **HIV p24 Antigen EIA Screening** (pos=1 ; neg=0) | | |
| 73 | | **HIV p24 Antigen Neutralization** (pos=1 ; neg=0) | | |
| 74 | | | | |
| 75 | | *The subsequent Inno-Lia data originate from:* | *this lab* | **Change** |
| 76 | **Inno-Lia HIV-1/2 Score** (enter all reactions including those negative with a score [0, .5, 1, 2, 3 or 4]) | sgp120 (HIV-1) | | **n.a.** |
| 77 | | gp41 (HIV-1) | | **n.a.** |
| 78 | | p31 | | **n.a.** |
| 79 | | p24 | | **n.a.** |
| 80 | | p17 | | **n.a.** |
| 81 | | sgp105 (HIV-2) | | **n.a.** |
| 82 | | gp36 (HIV-2) | | **n.a.** |

## 6. Overall case interpretation

This section provides an automated evaluation of all results entered into sections 1—5 with regard to the requirements of the SFOPH's HIV Testing Concept (see also Table 1).

An anonymized case code is automatically generated from the ID data of section 1. The Tool checks whether there was clear reactivity in at least 2 different types of HIV tests and whether two different specimens each exhibited a positive result. It states whether the infection is by HIV-1 or HIV-2 or both or whether the type could not be established clearly. Regarding HIV-1 group it states that although Group-M infection is most likely, positive identification can only be achieved by means of a genetic resistance test. If Group-O infection has indeed been demonstrated, this is of course stated clearly.

Regarding viral load, if HIV-1 RNA is below 1'000 copies/mL, the Tool requests that the concentration be confirmed by the sequence-independent PERT assay. It recommends that HIV-1 RNA between 1'000 and 10'000 copies/mL should also be confirmed by the PERT assay if the patient shows a rapid CD4+ T-cell decline or is already in an advanced stage of disease. If results of the PERT assay were entered and yielded a viral load more than 5 times higher than that found by HIV-1 RNA PCR, the Tool recommends that viral load be monitored by means of the PERT assay.

The Tool also decides whether the case is a recent infection (≤12 months), and with what probability (94 or 98%), or whether it was ruled older. It also states that genetic resistance testing should be done. Genetic resistance test comments can also be entered manually; thus it is possible to note HIV type, group and subtype identified. All automated decisions of the Tool can be manually overruled if they are deemed faulty — but this is only necessary in extremely rare, diagnostically complicated cases.

If all requirements of the HIV Testing Concept are met, the tool yields the answer "Yes"; otherwise the answer is "No" and the points requiring clarification will be seen below — the fields either remain empty or will show appropriate warnings (see the examples in the appendix).

| **Overall Case Interpretation** (combined data of 1st, 2nd & 3rd specimen) | | | **CASE:** |
|---|---|---|---|
| 95 | | **Meets ALL SFOPH 2006 requirements (YES / NO)** | |
| 96 | | Clear reactivity in >= 2 different types of HIV tests | |
| 97 | | Positive, non-discrepant results in >= 2 different samples | |
| 98 | | HIV Type (HIV-1 or/and HIV-2) | |
| 99 | | Likely HIV-1 Group (M or O) | |
| 100 | | **HIV-1** viral load, as by Roche's or Abbott's test(s) [copies/ml] | |
| 101 | | Viral Load, as by PERT Assay [copies/ml] | |
| 102 | | Comments regarding the plausibility of Viral Load (VL) | |
| 103 | | Likely duration of infection (applies only to HIV-1) | |
| 104 | | Genetic Resistance Testing (GRT) Comments | |
| 105 | **Confirmatory Lab's Own Comments:** | | |
| 106 | | | |

## 7. Letter of information to the treating physician

The second page of the case-sheet is formatted as a letter to the treating physician. It contains a brief description of the requirements of the HIV Testing Concept (in German, French, or Italian) and a summary of whether the requirements have been met (in the formate of the above-shown overall case interpretation). This letter may accompany intermediate result reports if deemed useful; it should at least be enclosed with the final result report.

## 8. Additional functions

The Tool contains functions (Visual Basic) for

- Opening a new case sheet using a template that can be customized by the user
- Closing a completed case, thereby
  - generating an e-mail notification containing all data in anonymized form to the SFOPH and the SNCR,
  - depositing a copy of that notification within the folder in which the Tool is located
  - generating an external protocol of all notifications mailed to the SFOPH
  - transferring all case data to a list of completed cases remaining within the tool

- generating a printed pre-addressed case notification that can be sent easily by post to the Cantonal Medical Officer (e-mail transfer also possible)
- deleting the case sheet of the completed case from the Tool. As a result, the Tool contains only the active case sheets and remains slim and easily manageable even after prolongued use.

**References**

1. Schupbach J, Bisset LR, Gebhardt MD, Regenass S, Burgisser P, et al. (2012) Diagnostic performance of line-immunoassay based algorithms for incident HIV-1 infection. BMC Infect Dis 12: 88. http://www.ncbi.nlm.nih.gov/pubmed/22497961?dopt=Citation

2. Schupbach J, Bisset LR, Regenass S, Burgisser P, Gorgievski M, et al. (2011) High specificity of line-immunoassay based algorithms for recent HIV-1 infection independent of viral subtype and stage of disease. BMC Infect Dis 11: 254. http://www.ncbi.nlm.nih.gov/pubmed/21943091?dopt=Citation

3. Schupbach J, Gebhardt MD, Tomasik Z, Niederhauser C, Yerly S, et al. (2007) Assessment of recent HIV-1 infection by a line immunoassay for HIV-1/2 confirmation. PLoS Med 4: e343. http://www.ncbi.nlm.nih.gov/pubmed/18052604?dopt=Citation

see also Fig. 4 on p. 28 of the document National Programme on HIV and other STI (NPHS) 2011-2017 dated 30.11.2010 | 3436 kb | PDF, which is downloadable from http://www.bag.admin.ch/hiv_aids/05464/05465/12491/index.html?lang=en

**Table 1. The four questions of HIV diagnosis, associated tasks, requirements and responsibilities**

| Question | Task | Requirements | Responsibilities |
|---|---|---|---|
| **1. Is someone infected with HIV?** | HIV screening at >18 months | CE-marked HIV-1/2/O antibody + antigen as a combination test for laboratories and as a rapid-test | SFOPH-accredited medical-diagnostic laboratories, physician's practices or HIV testing centers<br>HIV notification laboratories<br>SNCR |
| | Verification (confirmation) of diagnosis | Two different CE-marked tests are clearly reactive or positive<br>Two different samples have been tested, each with a clearly reactive or positive result | Collaboratively: SFOPH-accredited medical-diagnostic laboratories, physician's practices or HIV testing centers<br>HIV notification laboratories<br>SNCR |
| | Neonatal/Pediatric HIV screening at <18 months | CE-marked HIV-1/2/O antibody + antigen combination test to see whether the baby was exposed to maternal HIV infection<br>CE-marked HIV-1/2 Line Immunoassay to determine the correct test (HIV-1 or HIV-2) for initial testing; thereafter | HIV notification laboratories in Basel and Geneva, as well as the SNCR |
| | | For HIV-1: HIV-1 RNA in plasma<br>and/or<br>For HIV-2: HIV-2 DNA in PBMC by means of a high-input (MEGA) PCR | HIV notification laboratories in Basel and Geneva, as well as the SNCR<br>SNCR |
| **2. What properties does the virus have?** | Discrimination between HIV-1 and HIV-2, detection of double-infection with HIV-1 & HIV-2 | CE-marked HIV-1/2 Line Immunoassay | HIV notification laboratories<br>SNCR |
| | Confirmation of HIV-2 infection | High-input (MEGA) PCR for HIV-2 in PBMC | SNCR |
| | Resistance against antiretroviral medications | Genotypic resistance-testing of at least the PR and RT regions, optimally also the IN region | HIV notification laboratories in Basel, Geneva and Lausanne, as well as the SNCR<br>SNCR for HIV-2 and Group-O HIV-1 infections |
| | Identification of Group-O HIV-1 | Takes place most effectively via sequencing within the framework of genotypic resistance-testing<br>Group-O-specific DNA and RNA-PCR | SNCR; the HIV notification laboratories send their 'unamplifiable despite adequate HIV-1 viral load' isolates to the SNCR<br>SNCR |

(continued on next page)

| | | | |
|---|---|---|---|
| **3. How high is the viral load?** | HIV-1 quantification | CE-marked viral load tests for HIV-1 | SFOPH-accredited medical-diagnostic laboratories<br>HIV notification laboratories<br>SNCR |
| | HIV-2 quantification | Sequence-independent testing using the PERT assay | SNCR |
| | HIV-1 & HIV-2 double-infection | CE-labeled viral load test for HIV-1<br><br>HIV-2 RNA, qualitative or quantitative | HIV notification laboratories<br>SNCR<br><br>SNCR |
| | Verification of a viral load of <1,000 copies/mL obtained using a commercial kit | Sequence-independent testing using the PERT assay | SNCR |
| **4. How large is the proportion of recent infections among the newly reported HIV-infections?** | Calculation of the fraction of recent infections <12 months among all reported cases | Data already at hand from the HIV-1/2 line immunoassay | HIV notification laboratories<br>SNCR<br>SFOPH |
| Ambiguities and problems of any kind | | Depending on the problem | SNCR |

Abbreviations: PR, protease; RT, reverse transcriptase; IN, integrase; PBMC, peripheral blood mononuclear cells; SFOPH, Swiss Federal Office of Public Health; SNCR, Swiss National Center for Retroviruses

**Appendix**. Examples of cases at different steps of HIV confirmation using the Tool.

***Example 1:***

In this case, a first sample of a patient had shown a positive result in a rapid 4th Generation HIV-1/2 Ab+Ag Combination assay performed in a doctor's office. The Notification Lab received a new sample and performed a 4th generation HIV-1/2 Ab/Ag Combination EIA, which also yielded a positive result. Thus, the patient had positive results with 4th Generation Screening tests on each of two different samples.

The Overall Case Interpretation at this point of testing records that only one of all the different criteria of the HIV Testing Concept is met — all others are still unmet.

| | **Outside results (from clinical labs, doctors' offices, VCT centers)** | **RESULT** |
|---|---|---|
| 12 | **HIV-1/2 Rapid *Antibody Only* Assay Result** (pos=1 ; ind=0.5; neg=0) | |
| 13 | **HIV-1/2 Rapid *Ab+Ag Combo* Assay** (pos=1 ; ind=0.5; neg=0) | 1 |
| 14 | **4th Generation HIV-1/2 Ab/Ag EIA** (pos=1 ; ind=0.5; neg=0) | |
| 15 | **Were the above test(s) done on a sample different from that received by the CL? (YES/NO/UNKNOWN)** | YES |

| | **Tests done on the 1st sample received in the Confirmatory Lab** | | **RESULT** |
|---|---|---|---|
| 19 | | **4th Generation HIV-1/2 Ab/Ag Combination EIA; Test A** (pos=1 ; ind=0.5; neg=0) | 1 |
| 20 | | (Signal/Cutoff Ratio) | |
| 21 | | | |
| 22 | | **4th Generation HIV-1/2 Ab/Ag Combination EIA; Test B** (pos=1 ; ind=0.5; neg=0) | |
| 23 | | (Signal/Cutoff Ratio) | |
| 24 | | | |
| 25 | | **3rd Generation HIV-1/2 *Antibody-ONLY* EIA** (pos=1 ; neg=0) | |
| 26 | | (Signal/Cutoff Ratio) | |
| 27 | | | |
| 28 | | **HIV p24 Antigen EIA Screening** (pos=1 ; neg=0) | |
| 29 | | (Signal/Cutoff Ratio) | |
| 30 | | | |
| 31 | | **HIV p24 Antigen Neutralization** (pos=1 ; neg=0) | |
| 32 | | | |
| 33 | | *The subsequent Inno-Lia data originate from:* | *this lab* |
| 34 | **Inno-Lia HIV-1/2 Score, 1st Sample** (enter all reactions including those negative with a score [0, .5, 1, 2, 3 or 4]) | sgp120 (HIV-1) | |
| 35 | | gp41 (HIV-1) | |
| 36 | | p31 | |
| 37 | | p24 | |
| 38 | | p17 | |
| 39 | | sgp105 (HIV-2) | |
| 40 | | gp36 (HIV-2) | |
| 41–44 | | Inno-Lia HIV-1/2 Score Interpretation I | |
| 45–47 | | Type Differentiation | |
| 48–49 | | Likely recency status and % specificity if classified as RECENT *(applies only if HIV-1 infection is confirmed !)* | |

| | **Overall Case Interpretation** (combined data of 1st, 2nd & 3rd specimen) | **CASE:** -- |
|---|---|---|
| 95 | **Meets ALL SFOPH 2006 requirements (YES / NO)** | **NO — see details below.** |
| 96 | Clear reactivity in >= 2 different types of HIV tests | **NO** |
| 97 | Positive, non-discrepant results in >= 2 different samples | **YES — but note that some results are from a different lab!** |
| 98 | HIV Type (HIV-1 or/and HIV-2) | |
| 99 | Likely HIV-1 Group (M or O) | |
| 100 | **HIV-1** viral load, as by Roche's or Abbott's test(s) [copies/ml] | |
| 101 | Viral Load, as by PERT Assay [copies/ml] | |
| 102 | Comments regarding the plausibility of Viral Load (VL) | |
| 103 | Likely duration of infection (applies only to HIV-1) | |
| 104 | Genetic Resistance Testing (GRT) Comments | |
| 105–106 | **Confirmatory Lab's Own Comments:** | |

***Example 2:***

In this case, a first sample of a patient had shown a positive result in a rapid 4th Generation HIV-1/2 Ab+Ag Combination assay. The same sample was sent to the Notification Lab, which conducted an Inno-Lia HIV I/II Score assay. This test showed a low-positive result with a pattern of incomplete seroconversion suggestive of primary HIV-1 infection. The tool rules this sample a recent HIV-1 infection with 98% probability. The case was positive by two different tests — one of the two criteria for confirmation of an HIV infection is thus met. As the sample tested by the Notification Lab was the same as that tested in the outside lab, a second sample has to be tested to exclude sample mixup; viral load testing (as well as resistance testing) is also pending.

| Outside results (from clinical labs, doctors' offices, VCT centers) | | | RESULT |
|---|---|---|---|
| 12 | | HIV-1/2 Rapid *Antibody Only* Assay Result (pos=1 ; ind=0.5; neg=0) | |
| 13 | | HIV-1/2 Rapid *Ab+Ag Combo* Assay (pos=1 ; ind=0.5; neg=0) | 1 |
| 14 | | 4th Generation HIV-1/2 Ab/Ag EIA (pos=1 ; ind=0.5; neg=0) | |
| 15 | | Were the above test(s) done on a sample different from that received by the CL? (YES/NO/UNKNOWN) | NO |
| **Tests done on the 1st sample received in the Confirmatory Lab** | | | **RESULT** |
| 19 | | 4th Generation HIV-1/2 Ab/Ag Combination EIA; Test A (pos=1 ; ind=0.5; neg=0) | |
| 20 | | (Signal/Cutoff Ratio) | |
| 21 | | | |
| 22 | | 4th Generation HIV-1/2 Ab/Ag Combination EIA; Test B (pos=1 ; ind=0.5; neg=0) | |
| 23 | | (Signal/Cutoff Ratio) | |
| 24 | | | |
| 25 | | 3rd Generation HIV-1/2 *Antibody-ONLY* EIA (pos=1 ; neg=0) | |
| 26 | | (Signal/Cutoff Ratio) | |
| 27 | | | |
| 28 | | HIV p24 Antigen EIA Screening (pos=1 ; neg=0) | |
| 29 | | (Signal/Cutoff Ratio) | |
| 30 | | | |
| 31 | | HIV p24 Antigen Neutralization (pos=1 ; neg=0) | |
| 32 | | | |
| 33 | | *The subsequent Inno-Lia data originate from:* | *this lab* |
| 34 | **Inno-Lia HIV-1/2 Score, 1st Sample** (enter all reactions including those negative with a score [0, .5, 1, 2, 3 or 4]) | sgp120 (HIV-1) | 1 |
| 35 | | gp41 (HIV-1) | 2 |
| 36 | | p31 | 0 |
| 37 | | p24 | 1 |
| 38 | | p17 | 0 |
| 39 | | sgp105 (HIV-2) | 0 |
| 40 | | gp36 (HIV-2) | 0 |
| 41 | | Inno-Lia HIV-1/2 Score Interpretation I | |
| 42 | | | HIV-POS |
| 43 | | | |
| 44 | | | Low intensity! |
| 45 | | Type Differentiation | HIV-1 |
| 46 | | | |
| 47 | | | |
| 48 | | Likely recency status and % specificity if classified as RECENT (*applies only if HIV-1* | RECENT |
| 49 | | *infection is confirmed !*) | 98% |

| **Overall Case Interpretation** (combined data of 1st, 2nd & 3rd specimen) | | | **CASE:** -- |
|---|---|---|---|
| 95 | | **Meets ALL SFOPH 2006 requirements (YES / NO)** | **NO — see details below.** |
| 96 | | Clear reactivity in >= 2 different types of HIV tests | **YES** |
| 97 | | Positive, non-discrepant results in >= 2 different samples | **NO** |
| 98 | | HIV Type (HIV-1 or/and HIV-2) | **HIV-1** |
| 99 | | Likely HIV-1 Group (M or O) | **Probably M, but this will be known only after resistance testing !** |
| 100 | | **HIV-1** viral load, as by Roche's or Abbott's test(s) [copies/ml] | |
| 101 | | Viral Load, as by PERT Assay [copies/ml] | |
| 102 | | Comments regarding the plausibility of Viral Load (VL) | |
| 103 | | Likely duration of infection (applies only to HIV-1) | **Less than 12 months, with a probability of about 98%** |
| 104 | | Genetic Resistance Testing (GRT) Comments | GRT (PR+RT+IN) recommended for all newly diagnosed HIV-1 infections! |
| 105 | **Confirmatory Lab's Own Comments:** | | |
| 106 | | | |

## *Example 2B:*

Continuing with *Example 2*, a new specimen was received by the Notification Lab with the request to determine the viral load and to perform genetic resistance testing. With the new results entered, namely a HIV-1 RNA load of 1'500'000 copies/mL on Sample 2 and a successful resistance test, all requirements of the HIV Testing Concept are met, as shown in the Overall Case Interpretation below. GRT comment was inserted manually — all other comments were automatically generated.

Note also the warning that is displayed if the patient information in section 1 is incomplete.

**Outside results (from clinical labs, doctors' offices, VCT centers)**

| | | RESULT |
|---|---|---|
| 12 | HIV-1/2 Rapid *Antibody Only* Assay Result (pos=1 ; ind=0.5; neg=0) | |
| 13 | HIV-1/2 Rapid *Ab+Ag Combo* Assay (pos=1 ; ind=0.5; neg=0) | 1 |
| 14 | 4th Generation HIV-1/2 Ab/Ag EIA (pos=1 ; ind=0.5; neg=0) | |
| 15 | Were the above test(s) done on a sample different from that received by the CL? (YES/NO/UNKNOWN) | NO |

**Tests done on the 1st sample received in the Confirmatory Lab**

| | | | RESULT |
|---|---|---|---|
| 19 | | 4th Generation HIV-1/2 Ab/Ag Combination EIA; Test A (pos=1 ; ind=0.5; neg=0) | |
| 20 | | (Signal/Cutoff Ratio) | |
| 21 | | | |
| 22 | | 4th Generation HIV-1/2 Ab/Ag Combination EIA; Test B (pos=1 ; ind=0.5; neg=0) | |
| 23 | | (Signal/Cutoff Ratio) | |
| 24 | | | |
| 25 | | 3rd Generation HIV-1/2 *Antibody-ONLY* EIA (pos=1 ; neg=0) | |
| 26 | | (Signal/Cutoff Ratio) | |
| 27 | | | |
| 28 | | HIV p24 Antigen EIA Screening (pos=1 ; neg=0) | |
| 29 | | (Signal/Cutoff Ratio) | |
| 30 | | | |
| 31 | | HIV p24 Antigen Neutralization (pos=1 ; neg=0) | |
| 32 | | | |
| 33 | | *The subsequent Inno-Lia data originate from:* | *this lab* |
| 34 | **Inno-Lia HIV-1/2 Score, 1st Sample** (enter all reactions including those negative with a score [0, .5, 1, 2, 3 or 4]) | sgp120 (HIV-1) | 1 |
| 35 | | gp41 (HIV-1) | 2 |
| 36 | | p31 | 0 |
| 37 | | p24 | 1 |
| 38 | | p17 | 0 |
| 39 | | sgp105 (HIV-2) | 0 |
| 40 | | gp36 (HIV-2) | 0 |
| 41–44 | | Inno-Lia HIV-1/2 Score Interpretation I | HIV-POS<br>Low intensity! |
| 45–47 | | Type Differentiation | HIV-1 |
| 48–49 | | Likely recency status and % specificity if classified as RECENT (*applies only if HIV-1 infection is confirmed !*) | RECENT<br>98% |

**Commercial nucleic acid tests** (mark whether done on 1st or 2nd sample!)

| | | RESULT | Sample |
|---|---|---|---|
| 52 | ROCHE COBAS Taqman HIV-1 Monitor RNA Quantitative (copies/mL; undetectable = 0) | 1'500'000 | 2 |
| 53 | | | |
| 54 | Abbott real-time HIV-1 RNA Quantitative (copies/mL; undetectable = 0) | | |
| 55 | HIV-1 DNA qualitative (pos=1; neg=0) | | |

**Nucleic acid test results** (may be obtained from NZR; mark whether on 1st, 2nd, or 3rd sample)

| | | | |
|---|---|---|---|
| 57 | PERT Assay Quantitative (converted into HIV-1 copies/mL; undetectable = 0) | | |
| 58 | | | |
| 59 | HIV-2 SPECIFIC DNA Qualitative (pos=1; neg=0) | | |
| 60 | HIV-1 GROUP O-SPECIFIC DNA or RNA; Real-Time PCR (pos=1; neg=0) | | |
| 61 | HIV-1 GROUP M-SPECIFIC DNA or RNA; Real-Time PCR (pos=1; neg=0) | | |
| 62 | HIV-1 DNA, High Input (MEGA) Real-Time PCR, Qualitative (pos=1; neg=0) | | |
| 63 | HIV-2 DNA, High Input (MEGA) Real-Time PCR, Qualitative (pos=1; neg=0) | | |
| | | | Days difference 1st to 2nd sample |
| | Date of 2nd sample | | |

**Tests done on the 2nd or a 3rd sample received in the Confirmatory Lab**

| | | | | (Signal/Cutoff Ratio) |
|---|---|---|---|---|
| 67 | | 4th Generation HIV-1/2 Ab/Ag Combination EIA; Test A (pos=1 ; ind=0.5; neg=0) | | |
| 68 | | 4th Generation HIV-1/2 Ab/Ag Combination EIA; Test B (pos=1 ; ind=0.5; neg=0) | | |
| 69 | | | | |
| 70 | | 3rd Generation HIV-1/2 *Antibody-ONLY* EIA (pos=1 ; neg=0) | | |
| 71 | | | | |
| 72 | | HIV p24 Antigen EIA Screening (pos=1 ; neg=0) | | |
| 73 | | HIV p24 Antigen Neutralization (pos=1 ; neg=0) | | |
| 74 | | | | |
| 75 | | *The subsequent Inno-Lia data originate from:* | *this lab* | Change |
| 76 | **Inno-Lia HIV-1/2 Score** (enter all reactions including those negative with a score [0, .5, 1, 2, 3 or 4]) | sgp120 (HIV-1) | | n.a. |
| 77 | | gp41 (HIV-1) | | n.a. |
| 78 | | p31 | | n.a. |
| 79 | | p24 | | n.a. |
| 80 | | p17 | | n.a. |
| 81 | | sgp105 (HIV-2) | | n.a. |
| 82 | | gp36 (HIV-2) | | n.a. |
| 83–86 | | Inno-Lia HIV-1/2 Score Interpretation I | | |
| 87–89 | | Type Differentiation | | |
| 90–91 | | Likely recency status and % specificity if classified as RECENT (*applies only if HIV-1 infection is confirmed !*) | | |

*Patient ID fields incomplete!*

**Overall Case Interpretation** (combined data of 1st, 2nd & 3rd specimen) **CASE: 931653-XY-41803 / VD 1406 9999**

| | | |
|---|---|---|
| 95 | **Meets ALL SFOPH 2012 requirements (YES / NO)** | **YES** |
| 96 | Clear reactivity in >= 2 different types of HIV tests | **YES** |
| 97 | Positive, non-discrepant results in >= 2 different samples | **YES** |
| 98 | HIV Type (HIV-1 or/and HIV-2) | **HIV-1** |
| 99 | Likely HIV-1 Group (M or O) | **HIV-1 M** |
| 100 | HIV-1 viral load, as by Roche's or Abbott's test(s) [copies/ml] | **1'500'000** |
| 101 | Viral Load, as by PERT Assay [copies/ml] | |
| 102 | Comments regarding the plausibility of Viral Load (VL) | **(no comments)** |
| 103 | Likely duration of infection (applies only to HIV-1) | **Less than 12 months, with a probability of about 98%** |
| 104 | Genetic Resistance Testing (GRT) Comments | **GRT indicates HIV-1 / Group M / Subtype CRF02_AG** |
| 105 | **Confirmatory Lab's Own Comments:** | |
