# Supplementary material for: Decreasing Proportion of Recent Infections among Newly Diagnosed HIV-1 Cases in Switzerland, 2008 to 2013 Based on Line-Immunoassay-Based Algorithms
Source: PLoS One. 2015 Jul 31;10(7):e0131828. doi: 10.1371/journal.pone.0131828 (PMC4521810; doi:10.1371/journal.pone.0131828)
Supplement: S2 File — (PDF) [file pone.0131828.s004.pdf]

S2 File Assessment of assay sensitivity over time

This control assessment addresses the possibility that the decline in recent infection numbers could be due to an inadvertent increase in sensitivity of the Inno-Lia over time. Similarly, bands of given intensity might over the years be ruled to be of increasing strength. Both types of variation would manifest themselves as antibody reactions of increasingly higher average intensity. As a result, certain samples that would be ruled incident early in the course of the study might be ruled older when tested in later years.

We have checked this possible source of technical artifact by determining the average band intensity for all five antigens in the investigated cohort over time among recent and older infections. While there was some variation in both groups, there was no trend that could be correlated with the observed reduction of incident infections, and we found no difference that would explain the observed changes.

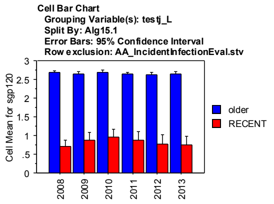

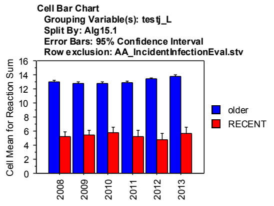

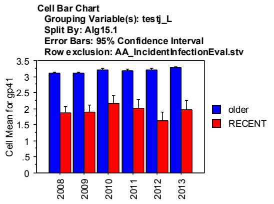

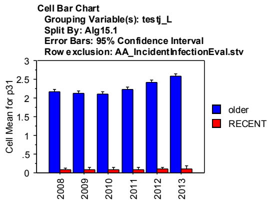

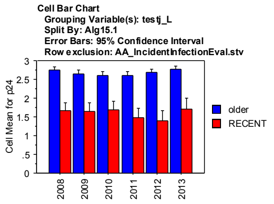

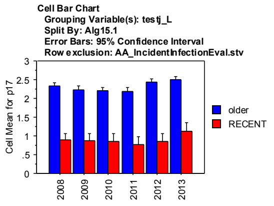

The column of figures on the left, from top to bottom, shows the average reaction to gp120, gp41, p31, p24, and p17 over the 6 years, among samples ruled recent (red) or older (blue) by the best-performing Alg15.1. The figure on the right shows the sum of the reactions to the five proteins for the same groups.

While there is some variation over time, there is no trend that could be correlated with the observed reduction of incident infections (shown below). Similar results were found for the other 9 algorithms used in the study (not shown). Thus, variation in test quality or result interpretation over time could not explain the observed decrease in recent infections.

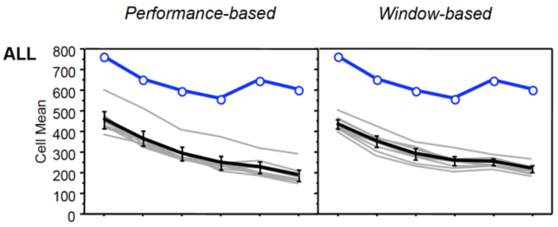

Thus, we conclude that the observed decrease in incident infections was not due to inadvertent change in the sensitivity of the Inno-Lia or in the interpretation of the band reactivity.
