# Supplementary material for: Decreasing Proportion of Recent Infections among Newly Diagnosed HIV-1 Cases in Switzerland, 2008 to 2013 Based on Line-Immunoassay-Based Algorithms
Source: PLoS One. 2015 Jul 31;10(7):e0131828. doi: 10.1371/journal.pone.0131828 (PMC4521810; doi:10.1371/journal.pone.0131828)
Supplement: S1 Table — (PDF) [file pone.0131828.s005.pdf]

S1 Table – Definition, diagnostic performance and window of the 10 best Inno-Lia algorithms for incident HIV-1

(combined from data published in Schüpbach J et al, BMC Infectious Diseases 2012, 12:88 and Schüpbach J et al, PLoS ONE, 8(8):e71662. doi:10.1371/journal.pone.0071662)

Alg #	Definition	% Sensitivity*	% Specificity	Performance Rank	Window (95% CI)
15.1	if (sgp120≤1 AND p31≤1 AND p17≤p24)<br>OR (gp41≤2 AND p31≤1 AND p17≤p24)<br>OR (p17 ≥ 2 AND p31=0 AND p17≤p24)<br>OR (p31=0 AND p24 ≥ 2 AND p17≤p24)<br>then RECENT<br>else older	31.9	95.14	1	120.3 (112.5—133.0)
15	if (sgp120≤1 AND p31≤1)<br>OR (gp41≤2 AND p31≤1)<br>OR (p17 ≥ 2 AND p31=0)<br>OR (p31=0 AND p24 ≥ 2)<br>then RECENT<br>else older	32.6	94.32	2	121.9 (103.0—160.0)
11.2	if (sgp120+gp41≤2.5)<br>OR ((sgp120+gp41+p31+p24+p17≤6.5) AND p31≤1 AND p17≤p24)<br>OR (p31=0 AND p24 ≥ 2)<br>then RECENT<br>else older	31.4	94.05	3.5	127.4 (109.0—162.0)
13	if (sgp120+gp41≤4 AND p31=0)<br>OR (p31=0 AND p24 ≥ 2)<br>then RECENT<br>else older	30.0	95.00	3.5	121.0 (99.5—178.0)
7	if sgp120+gp41+p31≤4<br>then RECENT<br>else older	24.6	98.38	5	105.3 (92.0—133.0)
9	if sgp120+gp41≤4 AND p31=0<br>then RECENT<br>else older	23.7	98.38	6	103.6 (80.0—250.0)
11.1	if (sgp120+gp41≤2.5)<br>OR ((sgp120+gp41+p31+p24+p17≤6.5) AND p31≤1<br>OR (p31=0 AND p24 ≥ 2)<br>then RECENT<br>else older	31.4	93.51	7	127.4 (109.0—162.0)

12.1	if (p24 ≥ 2 AND p31=0)<br>OR (gp41≤.5)<br>OR (sgp120+gp41+p31≤4)<br>OR (p31≤1 AND (sgp120+gp41+p31+p24+p17≤6.5))<br>then RECENT<br>else older	31.4	93.38	8	129.3 (106.0—186.0)
4.1	if p31≤0.5<br>then RECENT<br>else older	34.6	91.89	9	130.1 (94.0—570.0)
8.1	if gp41≤0.5<br>OR (sgp120+gp41+p31≤4)<br>OR ((sgp120+gp41+p31+p24+p17≤ 6.5) AND p31≤1)<br>then RECENT<br>else older	25.2	96.76	10	110.3 (90.0—176.0)

* Shown is sensitivity S1, which averages the sensitivities an algorithms exhibits in each of the four quarters of the 12-months incidence infection period; the underlying model assumes an even distribution of diagnosing incident HIV infection over all four quarters; Window in days; C.I., confidence interval.
