# Supplementary material for: Decreasing Proportion of Recent Infections among Newly Diagnosed HIV-1 Cases in Switzerland, 2008 to 2013 Based on Line-Immunoassay-Based Algorithms
Source: PLoS One. 2015 Jul 31;10(7):e0131828. doi: 10.1371/journal.pone.0131828 (PMC4521810; doi:10.1371/journal.pone.0131828)
Supplement: S2 Table — (PDF) [file pone.0131828.s006.pdf]

A. Calculation of performance-based incident infection rate (IIR-P) utilizing the adjusted sensitivities S1

ALG #	Diagnostic Performance: Sensitivity S1 (%)	Diagnostic Performance: Specificity (%)
15.1	31.90	95.14
15	32.60	94.32
11.2	31.40	94.05
13	30.00	95.00
7	24.60	98.38
9	23.70	98.38
11.1	31.40	93.37
12.1	31.40	93.38
4.1	34.60	91.89
8.1	25.20	96.76
mean	**29.68**	**95.07**
SD	3.78	2.17
lower limit 95%CI	*27.34*	*93.72*
upper limit 95%CI	*32.02*	*96.41*

ALG #	Notifications of 2008				
	Notifications with Inno-Lia; N=661				All; N=763
	N ruled older	N ruled incident	N estimated incident	IIR-P	Total N estimated incident
15.1	521	140	399	0.604	461
15	520	141	384	0.581	444
11.2	528	133	368	0.557	425
13	535	126	372	0.562	429
7	555	106	415	0.627	479
9	561	100	404	0.612	467
11.1	528	133	360	0.545	416
12.1	525	136	372	0.563	430
4.1	519	142	334	0.505	385
8.1	525	136	522	0.789	602
		mean	**393**	**0.595**	**454**
		SD	51	0.077	59
		lower limit 95%CI	*361*	*0.547*	*417*
		upper limit 95%CI	*425*	*0.642*	*490*

ALG #	Notifications of 2009					
	Notifications with Inno-Lia; N=599					All; N=649
	N ruled older	N ruled incident	N estimated incident	IIR	IIR, in % of baseline	Total N estimated incident
15.1	480	119	332	0.555	92.0	360
15	477	122	327	0.546	93.8	354
11.2	478	121	335	0.560	100.6	363
13	493	106	304	0.508	90.3	330
7	517	82	315	0.525	83.7	341
9	524	75	296	0.494	80.7	320
11.1	478	121	328	0.548	100.6	356
12.1	477	122	332	0.555	98.5	360
4.1	467	132	315	0.526	104.1	341
8.1	476	123	472	0.788	99.8	511
		mean	**336**	**0.560**	**94.4**	**364**
		SD	50	0.083	7.8	54
		lower limit 95%CI	*305*	*0.509*	*89.6*	*330*
		upper limit 95%CI	*366*	*0.612*	*99.2*	*397*

ALG #	Notifications of 2010					
	Notifications with Inno-Lia; N=587					All; N=597
	N ruled older	N ruled incident	N estimated incident	IIR	IIR, in % of baseline	Total N estimated incident
15.1	480	107	290	0.494	81.9	295
15	477	110	285	0.485	83.4	290
11.2	485	102	264	0.449	80.6	268
13	492	95	263	0.447	79.5	267
7	517	70	263	0.448	71.5	268
9	521	66	256	0.436	71.2	260
11.1	485	102	255	0.434	79.7	259
12.1	482	105	267	0.455	80.7	271
4.1	462	125	292	0.498	98.6	297
8.1	480	107	401	0.683	86.5	407
		mean	**283**	**0.483**	**81.4**	**288**
		SD	43	0.074	7.7	44
		lower limit 95%CI	*257*	*0.437*	*76.6*	*261*
		upper limit 95%CI	*310*	*0.529*	*86.1*	*316*

B. Calculation of windows-based incident infection rate (IIR-W)

ALG #	Window length and diagnostic specificity: Window length (d)	Window length and diagnostic specificity: Specificity (%)
15.1	120.3	95.14
15	121.9	94.32
11.2	127.4	94.05
13	121.0	95.00
7	105.3	98.38
9	103.6	98.38
11.1	127.4	93.37
12.1	129.3	93.38
4.1	130.1	91.89
8.1	110.3	96.76
mean	**119.7**	**95.07**
SD	9.9	2.17
lower limit 95%CI	*113.5*	*93.72*
upper limit 95%CI	*125.8*	*96.41*

ALG #	Notifications of 2008				
	Notifications with Inno-Lia; N=661				All; N=763
	N ruled older	N ruled incident	N estimated incident	IIR-W adjusted for specifity	Total N estimated incident
15.1	521	140	404	0.611	466
15	520	141	398	0.602	460
11.2	528	133	358	0.542	414
13	535	126	361	0.546	417
7	555	106	361	0.547	417
9	561	100	347	0.524	400
11.1	528	133	356	0.538	411
12.1	525	136	358	0.542	414
4.1	519	142	366	0.554	423
8.1	525	136	435	0.659	503
		mean	**375**	**0.567**	**432**
		SD	28	0.043	33
		lower limit 95%CI	*357*	*0.540*	*412*
		upper limit 95%CI	*392*	*0.593*	*453*

ALG #	Notifications of 2009					
	Notifications with Inno-Lia; N=599					All; N=649
	N ruled older	N ruled incident	N estimated incident	IIR-W adjusted for specifity	IIR, in % of baseline	Total N estimated incident
15.1	480	119	344	0.573	93.8	372
15	477	122	345	0.575	95.5	373
11.2	478	121	326	0.544	100.4	353
13	493	106	304	0.507	92.8	329
7	517	82	280	0.467	85.4	303
9	524	75	260	0.434	82.8	282
11.1	478	121	324	0.540	100.4	351
12.1	477	122	322	0.537	99.0	348
4.1	467	132	340	0.568	102.6	369
8.1	476	123	394	0.657	99.8	427
		mean	**324**	**0.540**	**95.2**	**351**
		SD	37	0.062	6.7	40
		lower limit 95%CI	*301*	*0.502*	*91.1*	*326*
		upper limit 95%CI	*347*	*0.579*	*99.4*	*376*

ALG #	Notifications of 2010					
	Notifications with Inno-Lia; N=587					All; N=597
	N ruled older	N ruled incident	N estimated incident	IIR-W adjusted for specifity	IIR, in % of baseline	Total N estimated incident
15.1	480	107	309	0.526	86.1	314
15	477	110	311	0.529	87.8	316
11.2	485	102	275	0.468	86.4	280
13	492	95	272	0.464	84.9	277
7	517	70	239	0.407	74.4	243
9	521	66	229	0.390	74.3	233
11.1	485	102	273	0.465	86.4	278
12.1	482	105	277	0.472	86.9	281
4.1	462	125	322	0.549	99.1	328
8.1	480	107	343	0.584	88.6	348
		mean	**285**	**0.485**	**85.5**	**290**
		SD	36	0.061	7.1	37
		lower limit 95%CI	*262*	*0.447*	*81.1*	*267*
		upper limit 95%CI	*307*	*0.523*	*89.9*	*312*

Notifications of 2011					
Notifications with Inno-Lia; N=549					All; N=557
N ruled older	N ruled incident	N estimated incident	IIR	IIR, in % of baseline	Total N estimated incident
462	87	223	0.406	67.3	226
456	93	230	0.418	71.9	233
455	94	241	0.439	78.8	245
468	81	214	0.390	69.4	217
485	64	240	0.437	69.6	243
491	58	222	0.405	66.2	226
455	94	233	0.424	77.8	236
453	96	241	0.439	77.9	244
451	98	202	0.368	72.8	205
450	99	370	0.674	85.3	375
	mean	**242**	**0.440**	**73.7**	**245**
	SD	47	0.085	6.1	47
	lower limit 95%CI	*213*	*0.387*	*69.9*	*216*
	upper limit 95%CI	*271*	*0.493*	*77.5*	*274*

Notifications of 2012					
Notifications with Inno-Lia; N=643					All; N=643
N ruled older	N ruled incident	N estimated incident	IIR	IIR, in % of baseline	Total N estimated incident
551	92	225	0.349	57.9	225
546	97	225	0.349	60.1	225
554	89	199	0.310	55.7	199
561	82	199	0.310	55.1	199
574	69	255	0.396	63.2	255
581	62	234	0.363	59.4	234
554	89	187	0.291	53.5	187
554	89	187	0.291	51.7	187
542	101	184	0.287	56.8	184
553	90	315	0.490	62.1	315
	mean	**221**	**0.344**	**57.5**	**221**
	SD	40	0.063	3.7	40
	lower limit 95%CI	*196*	*0.305*	*55.3*	*196*
	upper limit 95%CI	*246*	*0.383*	*59.8*	*246*

Notifications of 2013					
Notifications with Inno-Lia; N=597					All; N=600
N ruled older	N ruled incident	N estimated incident	IIR	IIR, in % of baseline	Total N estimated incident
519	78	181	0.303	50.3	182
516	81	175	0.293	50.4	176
519	78	167	0.280	50.2	168
527	70	161	0.269	47.8	161
540	57	206	0.345	55.0	207
545	52	192	0.321	52.5	193
519	78	155	0.260	47.7	156
518	79	159	0.267	47.4	160
510	87	146	0.244	48.3	146
515	82	285	0.478	60.5	287
	mean	**183**	**0.306**	**51.0**	**184**
	SD	40	0.068	4.1	41
	lower limit 95%CI	*158*	*0.264*	*48.5*	*158*
	upper limit 95%CI	*208*	*0.348*	*53.6*	*209*

All 3809 notifications, 2008–2013	
Total n estimated incident	Mean IIR
1749	0.459
1721	0.452
1668	0.438
1604	0.421
1792	0.471
1699	0.446
1609	0.422
1653	0.434
1559	0.409
2498	0.656
**1755**	**0.461**
270	0.071
1588	*0.417*
1923	*0.505*

Notifications of 2011					
Notifications with Inno-Lia; N=549					All; N=557
N ruled older	N ruled incident	N estimated incident	IIR-W adjusted for specifity	IIR, in % of baseline	Total N estimated incident
462	87	251	0.457	74.8	255
456	93	263	0.478	79.4	266
455	94	253	0.461	85.1	257
468	81	232	0.423	77.4	236
485	64	218	0.398	72.7	221
491	58	201	0.366	69.8	204
455	94	251	0.458	85.1	255
453	96	253	0.461	85.0	257
451	98	253	0.460	83.1	256
450	99	317	0.577	87.6	322
	mean	**249**	**0.454**	**80.0**	**253**
	SD	31	0.056	6.1	31
	lower limit 95%CI	*230*	*0.420*	*76.2*	*234*
	upper limit 95%CI	*268*	*0.489*	*83.8*	*272*

Notifications of 2012					
Notifications with Inno-Lia; N=643					All; N=643
N ruled older	N ruled incident	N estimated incident	IIR-W adjusted for specifity	IIR, in % of baseline	Total N estimated incident
551	92	266	0.413	67.6	266
546	97	274	0.426	70.7	274
554	89	240	0.373	68.8	240
561	82	235	0.365	66.9	235
574	69	235	0.366	66.9	235
581	62	215	0.334	63.7	215
554	89	238	0.370	68.8	238
554	89	235	0.365	67.3	235
542	101	260	0.405	73.1	260
553	90	288	0.448	68.0	288
	mean	**249**	**0.387**	**68.2**	**249**
	SD	22	0.035	2.5	22
	lower limit 95%CI	*235*	*0.365*	*66.6*	*235*
	upper limit 95%CI	*262*	*0.408*	*69.7*	*262*

Notifications of 2013					
Notifications with Inno-Lia; N=597					All; N=600
N ruled older	N ruled incident	N estimated incident	IIR-W adjusted for specifity	IIR, in % of baseline	Total N estimated incident
519	78	225	0.377	61.7	226
516	81	229	0.383	63.6	230
519	78	210	0.352	64.9	211
527	70	201	0.336	61.5	202
540	57	194	0.326	59.5	195
545	52	180	0.302	57.6	181
519	78	209	0.350	64.9	210
518	79	208	0.349	64.3	209
510	87	224	0.376	67.8	225
515	82	263	0.440	66.8	264
	mean	**214**	**0.359**	**63.3**	**215**
	SD	23	0.038	3.2	23
	lower limit 95%CI	*200*	*0.336*	*61.3*	*201*
	upper limit 95%CI	*228*	*0.382*	*65.2*	*229*

All 3809 notifications, 2008–2013	
Total n estimated incident	Mean IIR
1899	0.499
1919	0.504
1754	0.461
1695	0.445
1615	0.424
1514	0.398
1742	0.457
1744	0.458
1861	0.489
2151	0.565
**1790**	**0.470**
178	0.047
1679	*0.441*
1900	*0.499*
