# Supplementary material for: Decreasing Proportion of Recent Infections among Newly Diagnosed HIV-1 Cases in Switzerland, 2008 to 2013 Based on Line-Immunoassay-Based Algorithms
Source: PLoS One. 2015 Jul 31;10(7):e0131828. doi: 10.1371/journal.pone.0131828 (PMC4521810; doi:10.1371/journal.pone.0131828)
Supplement: S3 Table — (PDF) [file pone.0131828.s007.pdf]

S3 Table. Annual incident infection rates (IIR) according to likely mode of infection

1A. Calculation of performance-based incident infection rate (IIR-P) utilizing the adjusted sensitivities S1; HET

	Diagnostic Performance		Notifications of 2008					Notifications of 2009						Notifications of 2010						Notifications of 2011								
			Notifications with Inno-Lia; N=250				All; N=281	Notifications with Inno-Lia; N=240					All; N=253	Notifications with Inno-Lia; N=219					All; N=223	Notifications with Inno-Lia; N=212					All; N=213			
ALG #	Sensitivity S1 (%)	Specificity (%)	N ruled older	N ruled incident	N estimated incident	IIR-P	Total N estimated incident	N ruled older	N ruled incident	N estimated incident	IIR	IIR, in % of baseline	Total N estimated incident	N ruled older	N ruled incident	N estimated incident	IIR	IIR, in % of baseline	Total N estimated incident	N ruled older	N ruled incident	N estimated incident	IIR	IIR, in % of baseline	Total N estimated incident	N ruled older	N ruled incident	N estimated incident
15.1	31.9	95.14	211	39	99	0.397	112	200	40	105	0.437	109.9	110	196	23	46	0.209	52.5	47	188	24	51	0.239	60.2	51	203	27	59
15	32.6	94.32	211	39	92	0.368	104	200	40	98	0.408	110.8	103	195	24	43	0.196	53.2	44	185	27	56	0.262	71.1	56	202	28	55
11.2	31.4	94.05	212	38	91	0.363	102	201	39	97	0.405	111.4	102	199	20	27	0.125	34.4	28	184	28	60	0.285	78.5	61	205	25	44
13	30.0	95.00	216	34	86	0.344	97	206	34	88	0.367	106.6	93	200	19	32	0.147	42.7	33	188	24	54	0.253	73.5	54	207	23	46
7	24.6	98.38	226	24	87	0.347	98	214	26	96	0.401	115.5	101	207	12	37	0.168	48.4	37	194	18	63	0.299	86.1	64	213	17	58
9	23.7	98.38	226	24	90	0.361	102	218	22	82	0.342	94.6	86	208	11	34	0.154	42.6	34	194	18	66	0.311	86.1	66	215	15	51
11.1	31.4	93.37	212	38	86	0.346	97	201	39	93	0.388	112.3	98	199	20	22	0.101	29.2	23	184	28	56	0.266	76.8	57	205	25	39
12.1	31.4	93.38	212	38	87	0.346	97	201	39	93	0.389	112.2	98	198	21	26	0.120	34.6	27	184	28	56	0.266	76.8	57	205	25	39
4.1	34.6	91.89	208	42	82	0.328	92	202	38	70	0.292	88.9	74	194	25	27	0.125	38.0	28	182	30	48	0.228	69.5	49	203	27	32
8.1	25.2	96.76	222	28	91	0.362	102	213	27	88	0.365	100.6	92	206	13	27	0.123	33.9	27	190	22	69	0.325	89.7	69	213	17	43
mean	29.7	95.07		*mean*	89	0.356	100		*mean*	91	0.379	106.3	96		*mean*	32	0.147	41.0	33		*mean*	58	0.273	76.8	58		*mean*	47
SD	3.8	2.17		*SD*	5	0.019	5		*SD*	10	0.041	8.7	10		*SD*	8	0.035	8.3	8		*SD*	7	0.031	8.9	7		*SD*	9
lower limit 95%CI	27.3	93.72		*lower limit 95%CI*	86	0.345	97		*lower limit 95%CI*	85	0.354	100.9	90		*lower limit 95%CI*	27	0.125	35.8	28		*lower limit 95%CI*	54	0.254	71.3	54		*lower limit 95%CI*	41
upper limit 95%CI	32.0	96.41		*upper limit 95%CI*	92	0.368	103		*upper limit 95%CI*	97	0.404	111.7	102		*upper limit 95%CI*	37	0.169	46.1	38		*upper limit 95%CI*	62	0.293	82.3	62		*upper limit 95%CI*	52

1B. Calculation of windows-based incident infection rate (IIR-W); HET

	Window length and diagnostic specificity		Notifications of 2008					Notifications of 2009						Notifications of 2010						Notifications of 2011								
			Notifications with Inno-Lia; N=250				All; N=281	Notifications with Inno-Lia; N=240					All; N=253	Notifications with Inno-Lia; N=219					All; N=223	Notifications with Inno-Lia; N=212					All; N=213			
ALG #	Window length (d)	Specificity (%)	N ruled older	N ruled incident	N estimated incident	IIR-W adjusted for specifity	Total N estimated incident	N ruled older	N ruled incident	N estimated incident	IIR-W adjusted for specifity	IIR, in % of baseline	Total N estimated incident	N ruled older	N ruled incident	N estimated incident	IIR-W adjusted for specifity	IIR, in % of baseline	Total N estimated incident	N ruled older	N ruled incident	N estimated incident	IIR-W adjusted for specifity	IIR, in % of baseline	Total N estimated incident	N ruled older	N ruled incident	N estimated incident
15.1	120.3	95.14	211	39	113	0.450	127	200	40	115	0.481	106.8	122	196	23	66	0.303	67.3	68	188	24	69	0.327	72.6	70	203	27	78
15	121.9	94.32	211	39	110	0.441	124	200	40	113	0.471	106.8	119	195	24	68	0.309	70.2	69	185	27	76	0.360	81.6	77	202	28	79
11.2	127.4	94.05	212	38	102	0.410	115	201	39	105	0.438	106.9	111	199	20	54	0.246	60.1	55	184	28	75	0.356	86.9	76	205	25	67
13	121.0	95.00	216	34	97	0.390	110	206	34	97	0.406	104.2	103	200	19	54	0.249	63.8	55	188	24	69	0.324	83.2	69	207	23	66
7	105.3	98.38	226	24	82	0.327	92	214	26	89	0.369	112.8	93	207	12	41	0.187	57.1	42	194	18	61	0.290	88.4	62	213	17	58
9	103.6	98.38	226	24	83	0.333	94	218	22	76	0.318	95.5	80	208	11	38	0.174	52.3	39	194	18	62	0.294	88.4	63	215	15	52
11.1	127.4	93.37	212	38	102	0.407	114	201	39	104	0.435	106.9	110	199	20	54	0.244	60.1	54	184	28	75	0.353	86.9	75	205	25	67
12.1	129.3	93.38	212	38	100	0.401	113	201	39	103	0.428	106.9	108	198	21	55	0.253	63.1	56	184	28	74	0.348	86.9	74	205	25	66
4.1	130.1	91.89	208	42	108	0.433	122	202	38	98	0.408	94.2	103	194	25	64	0.294	67.9	66	182	30	77	0.365	84.2	78	203	27	70
8.1	110.3	96.76	222	28	90	0.359	101	213	27	86	0.360	100.4	91	206	13	42	0.190	53.0	42	190	22	70	0.332	92.7	71	213	17	54
mean	119.7	95.07		*mean*	99	0.395	111		*mean*	99	0.411	104.2	104		*mean*	54	0.245	61.5	55		*mean*	71	0.335	85.2	71		*mean*	66
SD	9.9	2.17		*SD*	11	0.043	12		*SD*	12	0.051	5.8	13		*SD*	11	0.049	6.1	11		*SD*	6	0.027	5.4	6		*SD*	9
lower limit 95%CI	113.5	93.72		*lower limit 95%CI*	92	0.368	103		*lower limit 95%CI*	91	0.380	100.6	96		*lower limit 95%CI*	47	0.215	57.7	48		*lower limit 95%CI*	68	0.318	81.8	68		*lower limit 95%CI*	60
upper limit 95%CI	125.8	96.41		*upper limit 95%CI*	105	0.422	118		*upper limit 95%CI*	106	0.443	107.7	112		*upper limit 95%CI*	60	0.275	65.3	61		*upper limit 95%CI*	74	0.351	88.5	75		*upper limit 95%CI*	71

2A. Calculation of performance-based incident infection rate (IIR-P) utilizing the adjusted sensitivities S1; IDU

	Diagnostic Performance		Notifications of 2008					Notifications of 2009						Notifications of 2010						Notifications of 2011								
			Notifications with Inno-Lia; N=20				All; N=24	Notifications with Inno-Lia; N=18					All; N=20	Notifications with Inno-Lia; N=12					All; N=12	Notifications with Inno-Lia; N=14					All; N=14			
ALG #	Sensitivity S1 (%)	Specificity (%)	N ruled older	N ruled incident	N estimated incident	IIR-P	Total N estimated incident	N ruled older	N ruled incident	N estimated incident	IIR	IIR, in % of baseline	Total N estimated incident	N ruled older	N ruled incident	N estimated incident	IIR	IIR, in % of baseline	Total N estimated incident	N ruled older	N ruled incident	N estimated incident	IIR	IIR, in % of baseline	Total N estimated incident	N ruled older	N ruled incident	N estimated incident
15.1	31.9	95.14	15	5	15	0.745	18	14	4	12	0.642	86.2	13	10	2	5	0.437	58.6	5	12	2	5	0.349	46.8	7	18	2	4
15	32.6	94.32	15	5	14	0.718	17	14	4	11	0.614	85.6	12	10	2	5	0.408	56.9	5	12	2	4	0.320	44.5	7	18	2	3
11.2	31.4	94.05	16	4	11	0.552	13	14	4	12	0.639	115.8	13	11	1	1	0.094	17.0	1	13	1	1	0.047	8.5	1	19	1	0
13	30.0	95.00	16	4	12	0.600	14	14	4	12	0.689	114.8	14	11	1	2	0.133	22.2	2	13	1	1	0.086	14.3	2	19	1	0
7	24.6	98.38	15	5	20	1.017	24	15	3	12	0.655	64.4	13	12	0	0	-0.070	0.0	0	14	0	0	-0.070	0.0	0	19	1	3
9	23.7	98.38	16	4	17	0.832	20	15	3	12	0.681	81.9	14	12	0	0	-0.073	0.0	0	14	0	0	-0.073	0.0	0	19	1	3
11.1	31.4	93.37	16	4	11	0.540	13	14	4	11	0.629	116.6	13	11	1	1	0.069	12.7	1	13	1	0	0.021	3.8	0	19	1	0
12.1	31.4	93.38	15	5	15	0.742	18	14	4	11	0.630	84.9	13	11	1	1	0.069	9.3	1	13	1	0	0.021	2.8	0	19	1	0
4.1	34.6	91.89	16	4	9	0.449	11	14	4	10	0.533	118.7	11	10	2	4	0.323	72.0	4	13	1	0	-0.037	0.0	0	18	2	1
8.1	25.2	96.76	15	5	20	0.991	24	15	3	11	0.611	61.7	12	12	0	0	-0.148	0.0	0	14	0	0	-0.148	0.0	0	19	1	2
mean	29.7	95.07		*mean*	14	0.719	17		*mean*	11	0.632	93.1	13		*mean*	2	0.124	24.9	2		*mean*	1	0.051	12.1	2		*mean*	2
SD	3.8	2.17		*SD*	4	0.190	5		*SD*	1	0.043	21.9	1		*SD*	2	0.204	27.3	2		*SD*	2	0.164	18.3	3		*SD*	2
lower limit 95%CI	27.3	93.72		*lower limit 95%CI*	12	0.601	14		*lower limit 95%CI*	11	0.606	79.5	12		*lower limit 95%CI*	1	-0.003	8.0	1		*lower limit 95%CI*	0	-0.050	0.7	0		*lower limit 95%CI*	1
upper limit 95%CI	32.0	96.41		*upper limit 95%CI*	17	0.836	20		*upper limit 95%CI*	12	0.659	106.6	13		*upper limit 95%CI*	3	0.251	41.8	3		*upper limit 95%CI*	2	0.153	23.4	4		*upper limit 95%CI*	3

2B. Calculation of windows-based incident infection rate (IIR-W)

	Window length and diagnostic specificity		Notifications of 2008					Notifications of 2009						Notifications of 2010						Notifications of 2011								
			Notifications with Inno-Lia; N=20				All; N=24	Notifications with Inno-Lia; N=18					All; N=20	Notifications with Inno-Lia; N=12					All; N=12	Notifications with Inno-Lia; N=14					All; N=14			
ALG #	Window length (d)	Specificity (%)	N ruled older	N ruled incident	N estimated incident	IIR-W adjusted for specifity	Total N estimated incident	N ruled older	N ruled incident	N estimated incident	IIR-W adjusted for specifity	IIR, in % of baseline	Total N estimated incident	N ruled older	N ruled incident	N estimated incident	IIR-W adjusted for specifity	IIR, in % of baseline	Total N estimated incident	N ruled older	N ruled incident	N estimated incident	IIR-W adjusted for specifity	IIR, in % of baseline	Total N estimated incident	N ruled older	N ruled incident	N estimated incident
15.1	120.3	95.14	15	5	14	0.722	17	14	4	12	0.641	88.9	13	10	2	6	0.481	66.7	6	12	2	6	0.412	57.1	9	18	2	6
15	121.9	94.32	15	5	14	0.706	17	14	4	11	0.628	88.9	13	10	2	6	0.471	66.7	6	12	2	6	0.403	57.1	8	18	2	6
11.2	127.4	94.05	16	4	11	0.539	13	14	4	11	0.599	111.1	12	11	1	3	0.225	41.7	3	13	1	3	0.192	35.7	4	19	1	3
13	121.0	95.00	16	4	11	0.573	14	14	4	11	0.637	111.1	13	11	1	3	0.239	41.7	3	13	1	3	0.205	35.7	4	19	1	3
7	105.3	98.38	15	5	17	0.853	20	15	3	10	0.568	66.7	11	12	0	0	0.000	0.0	0	14	0	0	0.000	0.0	0	19	1	3
9	103.6	98.38	16	4	14	0.693	17	15	3	10	0.578	83.3	12	12	0	0	0.000	0.0	0	14	0	0	0.000	0.0	0	19	1	3
11.1	127.4	93.37	16	4	11	0.535	13	14	4	11	0.594	111.1	12	11	1	3	0.223	41.7	3	13	1	3	0.191	35.7	4	19	1	3
12.1	129.3	93.38	15	5	13	0.659	16	14	4	11	0.586	88.9	12	11	1	3	0.220	33.3	3	13	1	3	0.188	28.6	4	19	1	3
4.1	130.1	91.89	16	4	10	0.516	12	14	4	10	0.573	111.1	11	10	2	5	0.430	83.3	5	13	1	3	0.184	35.7	4	18	2	5
8.1	110.3	96.76	15	5	16	0.800	19	15	3	10	0.534	66.7	11	12	0	0	0.000	0.0	0	14	0	0	0.000	0.0	0	19	1	3
mean	119.7	95.07		*mean*	13	0.660	16		*mean*	11	0.594	92.8	12		*mean*	3	0.229	37.5	3		*mean*	2	0.178	28.6	4		*mean*	4
SD	9.9	2.17		*SD*	2	0.117	3		*SD*	1	0.034	17.8	1		*SD*	2	0.189	30.0	2		*SD*	2	0.150	21.8	3		*SD*	1
lower limit 95%CI	113.5	93.72		*lower limit 95%CI*	12	0.587	14		*lower limit 95%CI*	10	0.573	81.8	11		*lower limit 95%CI*	1	0.112	18.9	1		*lower limit 95%CI*	1	0.085	15.0	2		*lower limit 95%CI*	3
upper limit 95%CI	125.8	96.41		*upper limit 95%CI*	15	0.732	18		*upper limit 95%CI*	11	0.615	103.8	12		*upper limit 95%CI*	4	0.346	56.1	4		*upper limit 95%CI*	4	0.271	42.1	6		*upper limit 95%CI*	5

ions of 2012			Notifications of 2013						All 1417 notifications, 2008–2013	
-Lia; N=230		All; N=230	Notifications with Inno-Lia; N=217					All; N=217		
IIR	IIR, in % of baseline	Total N estimated incident	N ruled older	N ruled incident	N estimated incident	IIR	IIR, in % of baseline	Total N estimated incident	Total n estimated incident	Total IIR
0.254	64.1	59	199	18	28	0.127	32.0	28	406	0.286
0.241	65.5	55	199	18	21	0.097	26.4	21	383	0.270
0.193	53.2	44	199	18	20	0.092	25.4	20	358	0.252
0.200	58.1	46	202	15	17	0.076	22.2	17	339	0.239
0.251	72.3	58	204	13	41	0.190	54.8	41	399	0.282
0.222	61.4	51	205	12	38	0.177	49.0	38	378	0.267
0.171	49.5	39	199	18	15	0.067	19.4	15	329	0.232
0.171	49.5	39	199	18	15	0.068	19.5	15	333	0.235
0.137	41.8	32	197	20	9	0.042	12.7	9	283	0.200
0.189	52.2	43	202	15	36	0.167	46.1	36	371	0.261
**0.203**	**56.7**	**47**		***mean***	**24**	**0.110**	**30.8**	**24**	**358**	**0.252**
0.039	9.2	9		*SD*	11	0.052	14.3	11	37	0.026
0.179	*51.1*	*41*		*lower limit 95%CI*	*17*	*0.078*	*21.9*	*17*	*334*	*0.236*
0.227	*62.4*	*52*		*upper limit 95%CI*	*31*	*0.143*	*39.6*	*31*	*381*	*0.269*

ions of 2012			Notifications of 2013						All 1417 notifications, 2008–2013	
-Lia; N=230		All; N=230	Notifications with Inno-Lia; N=217					All; N=217		
IIR-W adjusted for specifity	IIR, in % of baseline	Total N estimated incident	N ruled older	N ruled incident	N estimated incident	IIR-W adjusted for specifity	IIR, in % of baseline	Total N estimated incident	Total n estimated incident	Total IIR
0.339	75.3	78	199	18	52	0.239	53.2	52	515	0.364
0.344	78.0	79	199	18	51	0.234	53.2	51	518	0.366
0.293	71.5	67	199	18	49	0.224	54.6	49	472	0.333
0.287	73.5	66	202	15	43	0.198	50.8	43	446	0.315
0.252	77.0	58	204	13	44	0.204	62.4	44	391	0.276
0.226	67.9	52	205	12	42	0.192	57.6	42	369	0.260
0.291	71.5	67	199	18	48	0.222	54.6	48	469	0.331
0.287	71.5	66	199	18	47	0.219	54.6	47	465	0.328
0.303	69.9	70	197	20	52	0.238	54.9	52	489	0.345
0.237	66.0	54	202	15	48	0.221	61.7	48	408	0.288
**0.286**	**72.2**	**66**		***mean***	**48**	**0.219**	**55.7**	**48**	**454**	**0.321**
0.039	3.8	9		*SD*	4	0.016	3.7	4	51	0.036
0.262	*69.8*	*60*		*lower limit 95%CI*	*45*	*0.209*	*53.4*	*45*	*423*	*0.298*
0.310	*74.6*	*71*		*upper limit 95%CI*	*50*	*0.229*	*58.1*	*50*	*486*	*0.343*

ions of 2012			Notifications of 2013						All 102 notifications, 2008–2013	
-Lia; N=20		All; N=20	Notifications with Inno-Lia; N=12					All; N=12		
IIR	IIR, in % of baseline	Total N estimated incident	N ruled older	N ruled incident	N estimated incident	IIR	IIR, in % of baseline	Total N estimated incident	Total n estimated incident	Total IIR
0.190	25.5	5	10	2	5	0.437	58.6	5	53	0.522
0.160	22.4	4	10	2	5	0.408	56.9	5	50	0.491
-0.037	0.0	0	10	2	5	0.421	76.3	5	33	0.325
0.000	0.0	0	10	2	6	0.467	77.8	6	37	0.364
0.147	14.5	4	12	0	0	-0.070	0.0	0	41	0.404
0.153	18.4	4	12	0	0	-0.073	0.0	0	37	0.367
-0.066	0.0	0	10	2	5	0.405	75.1	5	32	0.310
-0.065	0.0	0	10	2	5	0.405	54.7	5	37	0.358
0.071	15.9	2	10	2	4	0.323	72.0	4	31	0.304
0.080	8.1	2	12	0	0	-0.148	0.0	0	38	0.373
**0.063**	**10.5**	**2**		***mean***	**3**	**0.257**	**47.1**	**3**	**39**	**0.382**
0.099	10.1	2		*SD*	2	0.248	33.6	2	7	0.073
0.002	*4.2*	*1*		*lower limit 95%CI*	*2*	*0.104*	*26.3*	*2*	*34*	*0.337*
0.125	*16.7*	*3*		*upper limit 95%CI*	*5*	*0.411*	*67.9*	*5*	*44*	*0.427*

ions of 2012			Notifications of 2013						All 102 notifications, 2008–2013	
-Lia; N=20		All; N=20	Notifications with Inno-Lia; N=12					All; N=12		
IIR-W adjusted for specifity	IIR, in % of baseline	Total N estimated incident	N ruled older	N ruled incident	N estimated incident	IIR-W adjusted for specifity	IIR, in % of baseline	Total N estimated incident	Total n estimated incident	Total IIR
0.289	40.0	7	10	2	6	0.481	66.7	6	58	0.564
0.282	40.0	7	10	2	6	0.471	66.7	6	56	0.552
0.135	25.0	3	10	2	5	0.449	83.3	5	40	0.396
0.143	25.0	4	10	2	6	0.478	83.3	6	43	0.421
0.171	20.0	4	12	0	0	0.000	0.0	0	36	0.354
0.173	25.0	4	12	0	0	0.000	0.0	0	33	0.319
0.134	25.0	3	10	2	5	0.446	83.3	5	40	0.393
0.132	20.0	3	10	2	5	0.439	66.7	5	43	0.419
0.258	50.0	6	10	2	5	0.430	83.3	5	44	0.436
0.160	20.0	4	12	0	0	0.000	0.0	0	34	0.332
**0.188**	**29.0**	**5**		***mean***	**4**	**0.319**	**53.3**	**4**	**43**	**0.419**
0.063	10.5	2		*SD*	3	0.221	37.5	3	8	0.083
0.148	*22.5*	*4*		*lower limit 95%CI*	*2*	*0.182*	*30.1*	*2*	*37*	*0.367*
0.227	*35.5*	*6*		*upper limit 95%CI*	*5*	*0.456*	*76.6*	*5*	*48*	*0.470*

3A. Calculation of performance-based incident infection rate (IIR-P) utilizing the adjusted sensitivities S1; MSM

	Diagnostic Performance		Notifications of 2008					Notifications of 2009						Notifications of 2010						Notifications of 2011								
			Notifications with Inno-Lia; N=227				All; N=268	Notifications with Inno-Lia; N=226					All; N=257	Notifications with Inno-Lia; N=248					All; N=254	Notifications with Inno-Lia; N=213					All; N=219			
ALG #	Sensitivity S1 (%)	Specificity (%)	N ruled older	N ruled incident	N estimated incident	IIR-P	Total N estimated incident	N ruled older	N ruled incident	N estimated incident	IIR	IIR, in % of baseline	Total N estimated incident	N ruled older	N ruled incident	N estimated incident	IIR	IIR, in % of baseline	Total N estimated incident	N ruled older	N ruled incident	N estimated incident	IIR	IIR, in % of baseline	Total N estimated incident	N ruled older	N ruled incident	N estimated incident
15.1	31.9	95.14	163	64	196	0.863	231	168	58	174	0.769	89.2	198	181	67	203	0.819	95.0	208	164	49	143	0.671	77.8	147	190	48	135
15	32.6	94.32	162	65	194	0.853	229	166	60	175	0.775	90.9	199	179	69	204	0.823	96.5	209	162	51	145	0.678	79.6	149	187	51	139
11.2	31.4	94.05	163	64	198	0.874	234	164	62	191	0.844	96.6	217	180	68	209	0.844	96.5	214	163	50	147	0.689	78.8	151	190	48	133
13	30.0	95.00	166	61	199	0.875	234	172	54	171	0.756	86.4	194	185	63	202	0.816	93.3	207	167	46	141	0.664	75.9	145	192	46	136
7	24.6	98.38	175	52	210	0.926	248	181	45	180	0.796	85.9	205	199	49	196	0.789	85.2	200	177	36	142	0.665	71.8	146	199	39	153
9	23.7	98.38	179	48	201	0.884	237	183	43	178	0.788	89.1	203	201	47	195	0.785	88.8	199	181	32	129	0.607	68.6	133	201	37	150
11.1	31.4	93.37	163	64	198	0.871	233	164	62	190	0.840	96.5	216	180	68	208	0.839	96.4	213	163	50	145	0.680	78.1	149	190	48	130
12.1	31.4	93.38	162	65	202	0.888	238	163	63	194	0.858	96.6	220	179	69	212	0.856	96.3	217	161	52	153	0.718	80.8	157	190	48	130
4.1	34.6	91.89	160	67	183	0.808	217	158	68	188	0.830	102.7	213	171	77	215	0.866	107.2	220	159	54	139	0.651	80.6	143	186	52	123
8.1	25.2	96.76	175	52	203	0.896	240	174	52	203	0.900	100.5	231	195	53	205	0.826	92.2	210	175	38	142	0.665	74.2	146	199	39	142
mean	29.7	95.07		*mean*	198	0.874	234		*mean*	184	0.816	93.4	210		*mean*	205	0.826	94.7	210		*mean*	142	0.669	76.6	146		*mean*	137
SD	3.8	2.17		*SD*	7	0.031	8		*SD*	10	0.046	5.9	12		*SD*	6	0.026	5.8	7		*SD*	6	0.028	4.0	6		*SD*	9
lower limit 95%CI	27.3	93.72		*lower limit 95%CI*	194	0.855	229		*lower limit 95%CI*	178	0.787	89.8	202		*lower limit 95%CI*	201	0.810	91.1	206		*lower limit 95%CI*	139	0.651	74.1	143		*lower limit 95%CI*	132
upper limit 95%CI	32.0	96.41		*upper limit 95%CI*	203	0.893	239		*upper limit 95%CI*	191	0.844	97.1	217		*upper limit 95%CI*	209	0.842	98.3	214		*upper limit 95%CI*	146	0.686	79.1	150		*upper limit 95%CI*	143

3B. Calculation of windows-based incident infection rate (IIR-W); MSM

	Window length and diagnostic specificity		Notifications of 2008					Notifications of 2009						Notifications of 2010						Notifications of 2011								
			Notifications with Inno-Lia; N=227				All; N=268	Notifications with Inno-Lia; N=226					All; N=257	Notifications with Inno-Lia; N=248					All; N=254	Notifications with Inno-Lia; N=213					All; N=219			
ALG #	Window length (d)	Specificity (%)	N ruled older	N ruled incident	N estimated incident	IIR-W adjusted for specifity	Total N estimated incident	N ruled older	N ruled incident	N estimated incident	IIR-W adjusted for specifity	IIR, in % of baseline	Total N estimated incident	N ruled older	N ruled incident	N estimated incident	IIR-W adjusted for specifity	IIR, in % of baseline	Total N estimated incident	N ruled older	N ruled incident	N estimated incident	IIR-W adjusted for specifity	IIR, in % of baseline	Total N estimated incident	N ruled older	N ruled incident	N estimated incident
15.1	120.3	95.14	163	64	185	0.814	218	168	58	167	0.741	91.0	190	181	67	193	0.780	95.8	198	164	49	141	0.664	81.6	145	190	48	139
15	121.9	94.32	162	65	184	0.809	217	166	60	169	0.750	92.7	193	179	69	195	0.786	97.2	200	162	51	144	0.676	83.6	148	187	51	144
11.2	127.4	94.05	163	64	172	0.760	204	164	62	167	0.739	97.3	190	180	68	183	0.739	97.3	188	163	50	135	0.633	83.3	139	190	48	129
13	121.0	95.00	166	61	175	0.770	206	172	54	155	0.685	88.9	176	185	63	181	0.728	94.5	185	167	46	132	0.619	80.4	136	192	46	132
7	105.3	98.38	175	52	177	0.781	209	181	45	153	0.679	86.9	175	199	49	167	0.674	86.3	171	177	36	123	0.576	73.8	126	199	39	133
9	103.6	98.38	179	48	166	0.733	196	183	43	149	0.659	90.0	169	201	47	163	0.657	89.6	167	181	32	111	0.521	71.0	114	201	37	128
11.1	127.4	93.37	163	64	171	0.754	202	164	62	166	0.734	97.3	189	180	68	182	0.733	97.3	186	163	50	134	0.628	83.3	138	190	48	128
12.1	129.3	93.38	162	65	171	0.755	202	163	63	166	0.735	97.4	189	179	69	182	0.733	97.2	186	161	52	137	0.644	85.3	141	190	48	127
4.1	130.1	91.89	160	67	173	0.761	204	158	68	175	0.776	101.9	199	171	77	199	0.800	105.2	203	159	54	139	0.654	85.9	143	186	52	134
8.1	110.3	96.76	175	52	167	0.733	197	174	52	167	0.737	100.4	189	195	53	170	0.684	93.3	174	175	38	122	0.571	77.9	125	199	39	125
mean	119.7	95.07		*mean*	174	0.767	206		*mean*	163	0.723	94.4	186		*mean*	181	0.731	95.4	186		*mean*	132	0.619	80.6	135		*mean*	132
SD	9.9	2.17		*SD*	6	0.028	7		*SD*	8	0.036	5.2	9		*SD*	12	0.049	5.1	12		*SD*	10	0.048	4.9	11		*SD*	6
lower limit 95%CI	113.5	93.72		*lower limit 95%CI*	170	0.750	201		*lower limit 95%CI*	158	0.701	91.2	180		*lower limit 95%CI*	174	0.701	92.2	178		*lower limit 95%CI*	125	0.588	77.5	129		*lower limit 95%CI*	128
upper limit 95%CI	125.8	96.41		*upper limit 95%CI*	178	0.784	210		*upper limit 95%CI*	169	0.746	97.6	192		*upper limit 95%CI*	189	0.762	98.5	193		*upper limit 95%CI*	138	0.649	83.7	142		*upper limit 95%CI*	136

4A. Calculation of performance-based incident infection rate (IIR-P) utilizing the adjusted sensitivities S1; UNKNOWN

	Diagnostic Performance		Notifications of 2008					Notifications of 2009						Notifications of 2010						Notifications of 2011								
			Notifications with Inno-Lia; N=161				All; N=186	Notifications with Inno-Lia; N=108					All; N=112	Notifications with Inno-Lia; N=105					All; N=105	Notifications with Inno-Lia; N=106					All; N=107			
ALG #	Sensitivity S1 (%)	Specificity (%)	N ruled older	N ruled incident	N estimated incident	IIR-P	Total N estimated incident	N ruled older	N ruled incident	N estimated incident	IIR	IIR, in % of baseline	Total N estimated incident	N ruled older	N ruled incident	N estimated incident	IIR	IIR, in % of baseline	Total N estimated incident	N ruled older	N ruled incident	N estimated incident	IIR	IIR, in % of baseline	Total N estimated incident	N ruled older	N ruled incident	N estimated incident
15.1	31.9	95.14	129	32	89	0.555	103	91	17	43	0.402	72.5	45	90	15	37	0.349	62.8	37	94	12	25	0.239	43.0	26	138	14	24
15	32.6	94.32	129	32	85	0.527	98	90	18	44	0.408	77.4	46	90	15	34	0.320	60.6	34	93	13	26	0.245	46.4	26	137	15	24
11.2	31.4	94.05	134	27	68	0.425	79	93	15	34	0.312	73.4	35	92	13	27	0.253	59.4	27	91	15	34	0.322	75.8	34	138	14	19
13	30.0	95.00	134	27	76	0.471	88	94	14	34	0.319	67.7	36	93	12	27	0.257	54.6	27	96	10	19	0.177	37.7	19	141	11	14
7	24.6	98.38	136	25	97	0.605	113	100	8	27	0.252	41.6	28	96	9	32	0.302	50.0	32	96	10	36	0.340	56.2	36	140	12	42
9	23.7	98.38	137	24	97	0.602	112	101	7	24	0.220	36.6	25	97	8	29	0.272	45.2	29	98	8	28	0.268	44.6	29	143	9	30
11.1	31.4	93.37	134	27	66	0.409	76	93	15	32	0.293	71.6	33	92	13	24	0.232	56.7	24	91	15	32	0.304	74.2	32	138	14	16
12.1	31.4	93.38	133	28	70	0.435	81	93	15	32	0.293	67.5	33	91	14	28	0.271	62.3	28	91	15	32	0.304	69.9	33	138	14	16
4.1	34.6	91.89	132	29	60	0.374	70	87	21	46	0.428	114.5	48	84	21	47	0.449	120.1	47	93	13	17	0.157	42.0	17	133	19	25
8.1	25.2	96.76	136	25	90	0.560	104	100	8	20	0.190	33.9	21	95	10	30	0.286	51.1	30	93	13	44	0.411	73.4	44	140	12	32
mean	29.7	95.07		*mean*	80	0.496	92		*mean*	34	0.312	65.7	35		*mean*	31	0.299	62.3	31		*mean*	29	0.277	56.3	30		*mean*	24
SD	3.8	2.17		*SD*	14	0.084	16		*SD*	9	0.081	23.8	9		*SD*	7	0.063	21.1	7		*SD*	8	0.076	15.4	8		*SD*	9
lower limit 95%CI	27.3	93.72		*lower limit 95%CI*	72	0.444	83		*lower limit 95%CI*	28	0.262	50.9	29		*lower limit 95%CI*	27	0.260	49.2	27		*lower limit 95%CI*	24	0.229	46.8	25		*lower limit 95%CI*	19
upper limit 95%CI	32.0	96.41		*upper limit 95%CI*	88	0.548	102		*upper limit 95%CI*	39	0.362	80.4	41		*upper limit 95%CI*	35	0.338	75.4	35		*upper limit 95%CI*	34	0.324	65.9	35		*upper limit 95%CI*	29

4B. Calculation of windows-based incident infection rate (IIR-W); UNKNOWN

	Window length and diagnostic specificity		Notifications of 2008					Notifications of 2009						Notifications of 2010						Notifications of 2011								
			Notifications with Inno-Lia; N=161				All; N=186	Notifications with Inno-Lia; N=108					All; N=112	Notifications with Inno-Lia; N=105					All; N=105	Notifications with Inno-Lia; N=106					All; N=107			
ALG #	Window length (d)	Specificity (%)	N ruled older	N ruled incident	N estimated incident	IIR-W adjusted for specifity	Total N estimated incident	N ruled older	N ruled incident	N estimated incident	IIR-W adjusted for specifity	IIR, in % of baseline	Total N estimated incident	N ruled older	N ruled incident	N estimated incident	IIR-W adjusted for specifity	IIR, in % of baseline	Total N estimated incident	N ruled older	N ruled incident	N estimated incident	IIR-W adjusted for specifity	IIR, in % of baseline	Total N estimated incident	N ruled older	N ruled incident	N estimated incident
15.1	120.3	95.14	129	32	92	0.574	107	91	17	49	0.454	79.2	51	90	15	43	0.412	71.9	43	94	12	35	0.327	57.0	35	138	14	40
15	121.9	94.32	129	32	90	0.561	104	90	18	51	0.471	83.9	53	90	15	42	0.403	71.9	42	93	13	37	0.346	61.7	37	137	15	42
11.2	127.4	94.05	134	27	73	0.452	84	93	15	40	0.374	82.8	42	92	13	35	0.334	73.8	35	91	15	40	0.381	84.4	41	138	14	38
13	121.0	95.00	134	27	77	0.481	89	94	14	40	0.371	77.3	42	93	12	34	0.328	68.1	34	96	10	29	0.270	56.3	29	141	11	32
7	105.3	98.38	136	25	85	0.530	98	100	8	27	0.253	47.7	28	96	9	31	0.292	55.2	31	96	10	34	0.322	60.8	34	140	12	41
9	103.6	98.38	137	24	83	0.517	96	101	7	24	0.225	43.5	25	97	8	28	0.264	51.1	28	98	8	28	0.262	50.6	28	143	9	31
11.1	127.4	93.37	134	27	72	0.449	83	93	15	40	0.372	82.8	42	92	13	35	0.331	73.8	35	91	15	40	0.379	84.4	41	138	14	37
12.1	129.3	93.38	133	28	74	0.458	85	93	15	40	0.366	79.9	41	91	14	37	0.351	76.7	37	91	15	40	0.373	81.4	40	138	14	37
4.1	130.1	91.89	132	29	75	0.464	86	87	21	54	0.501	108.0	56	84	21	54	0.516	111.0	54	93	13	34	0.316	68.1	34	133	19	49
8.1	110.3	96.76	136	25	80	0.497	92	100	8	26	0.237	47.7	27	95	10	32	0.305	61.3	32	93	13	42	0.393	79.0	42	140	12	38
mean	119.7	95.07		*mean*	80	0.498	93		*mean*	39	0.362	73.3	41		*mean*	37	0.354	71.5	37		*mean*	36	0.337	68.3	36		*mean*	39
SD	9.9	2.17		*SD*	7	0.046	8		*SD*	11	0.098	20.5	11		*SD*	8	0.073	16.3	8		*SD*	5	0.046	12.9	5		*SD*	5
lower limit 95%CI	113.5	93.72		*lower limit 95%CI*	76	0.470	87		*lower limit 95%CI*	33	0.302	60.6	34		*lower limit 95%CI*	32	0.308	61.4	32		*lower limit 95%CI*	33	0.308	60.4	33		*lower limit 95%CI*	35
upper limit 95%CI	125.8	96.41		*upper limit 95%CI*	85	0.526	98		*upper limit 95%CI*	46	0.423	86.0	47		*upper limit 95%CI*	42	0.399	81.6	42		*upper limit 95%CI*	39	0.365	76.3	39		*upper limit 95%CI*	42

ions of 2012			Notifications of 2013						All 1437 notifications, 2008–2013	
-Lia; N=238		All; N=238	Notifications with Inno-Lia; N=199					All; N=201		
IIR	IIR, in % of baseline	Total N estimated incident	N ruled older	N ruled incident	N estimated incident	IIR	IIR, in % of baseline	Total N estimated incident	Total n estimated incident	Total IIR
0.566	65.6	135	155	44	127	0.638	73.9	128	1047	0.729
0.585	68.6	139	153	46	129	0.648	76.0	130	1055	0.734
0.559	63.9	133	156	43	122	0.615	70.4	124	1073	0.747
0.573	65.5	136	158	41	124	0.624	71.3	125	1043	0.726
0.643	69.4	153	168	31	121	0.607	65.6	122	1074	0.747
0.631	71.3	150	171	28	112	0.564	63.8	113	1035	0.721
0.547	62.8	130	156	43	120	0.605	69.5	122	1063	0.740
0.547	61.5	130	155	44	124	0.625	70.4	126	1089	0.758
0.519	64.2	123	151	48	120	0.604	74.8	121	1037	0.722
0.599	66.8	142	168	31	112	0.562	62.7	113	1082	0.753
**0.577**	**66.0**	**137**		***mean***	**121**	**0.609**	**69.8**	**122**	**1060**	**0.738**
0.039	3.1	9		*SD*	6	0.028	4.6	6	19	0.013
0.553	*64.1*	*132*		*lower limit 95%CI*	*118*	*0.592*	*67.0*	*119*	*1048*	*0.729*
0.601	*67.9*	*143*		*upper limit 95%CI*	*125*	*0.627*	*72.7*	*126*	*1072*	*0.746*

ions of 2012			Notifications of 2013						All 1437 notifications, 2008–2013	
-Lia; N=238		All; N=238	Notifications with Inno-Lia; N=199					All; N=201		
IIR-W adjusted for specifity	IIR, in % of baseline	Total N estimated incident	N ruled older	N ruled incident	N estimated incident	IIR-W adjusted for specifity	IIR, in % of baseline	Total N estimated incident	Total n estimated incident	Total IIR
0.582	71.5	139	155	44	127	0.638	78.4	128	1019	0.709
0.605	74.8	144	153	46	130	0.653	80.7	131	1032	0.718
0.543	71.5	129	156	43	116	0.582	76.6	117	966	0.672
0.554	71.9	132	158	41	117	0.590	76.7	119	953	0.663
0.559	71.5	133	168	31	106	0.531	68.0	107	921	0.641
0.539	73.5	128	171	28	97	0.488	66.5	98	873	0.608
0.540	71.5	128	156	43	115	0.578	76.6	116	959	0.667
0.532	70.4	127	155	44	116	0.583	77.2	117	962	0.669
0.563	74.0	134	151	48	124	0.622	81.7	125	1009	0.702
0.525	71.5	125	168	31	99	0.499	68.0	100	910	0.633
**0.554**	**72.2**	**132**		***mean***	**115**	**0.576**	**75.1**	**116**	**960**	**0.668**
0.025	1.4	6		*SD*	11	0.056	5.5	11	50	0.035
0.539	*71.4*	*128*		*lower limit 95%CI*	*108*	*0.542*	*71.7*	*109*	*929*	*0.647*
0.569	*73.1*	*136*		*upper limit 95%CI*	*122*	*0.611*	*78.5*	*123*	*992*	*0.690*

ions of 2012			Notifications of 2013						All 826 notifications, 2008–2013	
-Lia; N=152		All; N=152	Notifications with Inno-Lia; N=163					All; N=164		
IIR	IIR, in % of baseline	Total N estimated incident	N ruled older	N ruled incident	N estimated incident	IIR	IIR, in % of baseline	Total N estimated incident	Total n estimated incident	Total IIR
0.161	29.0	24	149	14	22	0.138	24.8	23	258	0.312
0.156	29.5	24	148	15	21	0.131	24.8	21	249	0.301
0.128	30.1	19	148	15	21	0.128	30.1	21	215	0.261
0.089	19.0	14	151	12	15	0.094	20.1	15	198	0.240
0.273	45.1	42	150	13	45	0.277	45.7	45	296	0.358
0.195	32.4	30	151	12	42	0.260	43.2	43	266	0.322
0.104	25.4	16	148	15	17	0.104	25.4	17	199	0.241
0.105	24.1	16	148	15	17	0.104	24.0	17	208	0.251
0.166	44.3	25	146	17	14	0.088	23.4	14	221	0.267
0.212	37.9	32	148	15	44	0.272	48.5	45	276	0.334
**0.159**	**31.7**	**24**		***mean***	**26**	**0.159**	**31.0**	**26**	**239**	**0.289**
0.057	8.5	9		*SD*	13	0.078	10.6	13	35	0.042
0.124	*26.4*	*19*		*lower limit 95%CI*	*18*	*0.111*	*24.4*	*18*	*217*	*0.263*
0.194	*37.0*	*29*		*upper limit 95%CI*	*34*	*0.208*	*37.6*	*34*	*260*	*0.315*

ions of 2012			Notifications of 2013						All 826 notifications, 2008–2013	
-Lia; N=152		All; N=152	Notifications with Inno-Lia; N=163					All; N=164		
IIR-W adjusted for specifity	IIR, in % of baseline	Total N estimated incident	N ruled older	N ruled incident	N estimated incident	IIR-W adjusted for specifity	IIR, in % of baseline	Total N estimated incident	Total n estimated incident	Total IIR
0.266	46.3	40	149	14	40	0.248	43.2	41	317	0.384
0.279	49.7	42	148	15	42	0.260	46.3	43	322	0.389
0.248	54.9	38	148	15	40	0.248	54.9	41	280	0.339
0.207	43.2	32	151	12	34	0.211	43.9	35	260	0.315
0.269	50.8	41	150	13	44	0.272	51.4	45	277	0.336
0.205	39.7	31	151	12	42	0.255	49.4	42	250	0.303
0.246	54.9	37	148	15	40	0.246	54.9	40	278	0.337
0.243	53.0	37	148	15	40	0.243	52.9	40	280	0.339
0.322	69.4	49	146	17	44	0.269	57.9	44	324	0.392
0.253	50.8	38	148	15	48	0.295	59.3	48	280	0.339
**0.254**	**51.3**	**39**		***mean***	**42**	**0.255**	**51.4**	**42**	**287**	**0.347**
0.034	8.1	5		*SD*	4	0.022	5.6	4	25	0.031
0.233	*46.3*	*35*		*lower limit 95%CI*	*39*	*0.241*	*47.9*	*40*	*271*	*0.328*
0.275	*56.3*	*42*		*upper limit 95%CI*	*44*	*0.268*	*54.9*	*44*	*303*	*0.366*
