# Supplementary material for: Decreasing Proportion of Recent Infections among Newly Diagnosed HIV-1 Cases in Switzerland, 2008 to 2013 Based on Line-Immunoassay-Based Algorithms
Source: PLoS One. 2015 Jul 31;10(7):e0131828. doi: 10.1371/journal.pone.0131828 (PMC4521810; doi:10.1371/journal.pone.0131828)
Supplement: S4 Table — (PDF) [file pone.0131828.s008.pdf]

S4 Table. Annual incident infection estimates for the 3'809 HIV-1 notifications of 2008—2013; performance-based estimation; comparison of sensitivities S1, S2, and S3

A. Calculation of performance-based incident infection rate (IIR-P) utilizing the adjusted sensitivities S1

	Diagnostic Performance	
ALG #	Sensitivity S1 (%)	Specificity (%)
15.1	31.90	95.14
15	32.60	94.32
11.2	31.40	94.05
13	30.00	95.00
7	24.60	98.38
9	23.70	98.38
11.1	31.40	93.37
12.1	31.40	93.38
4.1	34.60	91.89
8.1	25.20	96.76
mean	**29.68**	**95.07**
SD	3.78	2.17
lower limit 95%CI	*27.34*	*93.72*
upper limit 95%CI	*32.02*	*96.41*

Notifications of 2008				
Notifications with Inno-Lia; N=661				All; N=763
N ruled older	N ruled incident	N estimated incident	IIR-P	Total N estimated incident
521	140	399	0.604	461
520	141	384	0.581	444
528	133	368	0.557	425
535	126	372	0.562	429
555	106	415	0.627	479
561	100	404	0.612	467
528	133	360	0.545	416
525	136	372	0.563	430
519	142	334	0.505	385
525	136	522	0.789	602
	mean	**393**	**0.595**	**454**
	SD	51	0.077	59
	lower limit 95%CI	*361*	*0.547*	*417*
	upper limit 95%CI	*425*	*0.642*	*490*

Notifications of 2009					
Notifications with Inno-Lia; N=599					All; N=649
N ruled older	N ruled incident	N estimated incident	IIR	IIR, in % of baseline	Total N estimated incident
480	119	332	0.555	92.0	360
477	122	327	0.546	93.8	354
478	121	335	0.560	100.6	363
493	106	304	0.508	90.3	330
517	82	315	0.525	83.7	341
524	75	296	0.494	80.7	320
478	121	328	0.548	100.6	356
477	122	332	0.555	98.5	360
467	132	315	0.526	104.1	341
476	123	472	0.788	99.8	511
	mean	**336**	**0.560**	**94.4**	**364**
	SD	50	0.083	7.8	54
	lower limit 95%CI	*305*	*0.509*	*89.6*	*330*
	upper limit 95%CI	*366*	*0.612*	*99.2*	*397*

Notifications of 2010					
Notifications with Inno-Lia; N=587					All; N=597
N ruled older	N ruled incident	N estimated incident	IIR	IIR, in % of baseline	Total N estimated incident
480	107	290	0.494	81.9	295
477	110	285	0.485	83.4	290
485	102	264	0.449	80.6	268
492	95	263	0.447	79.5	267
517	70	263	0.448	71.5	268
521	66	256	0.436	71.2	260
485	102	255	0.434	79.7	259
482	105	267	0.455	80.7	271
462	125	292	0.498	98.6	297
480	107	401	0.683	86.5	407
	mean	**283**	**0.483**	**81.4**	**288**
	SD	43	0.074	7.7	44
	lower limit 95%CI	*257*	*0.437*	*76.6*	*261*
	upper limit 95%CI	*310*	*0.529*	*86.1*	*316*

Notifications of 2011					
Notifications with Inno-Lia; N=549					All; N=557
N ruled older	N ruled incident	N estimated incident	IIR	IIR, in % of baseline	Total N estimated incident
462	87	223	0.406	67.3	226
456	93	230	0.418	71.9	233
455	94	241	0.439	78.8	245
468	81	214	0.390	69.4	217
485	64	240	0.437	69.6	243
491	58	222	0.405	66.2	226
455	94	233	0.424	77.8	236
453	96	241	0.439	77.9	244
451	98	202	0.368	72.8	205
450	99	370	0.674	85.3	375
	mean	**242**	**0.440**	**73.7**	**245**
	SD	47	0.085	6.1	47
	lower limit 95%CI	*213*	*0.387*	*69.9*	*216*
	upper limit 95%CI	*271*	*0.493*	*77.5*	*274*

B. Calculation of performance-based incident infection rate (IIR-P) utilizing the adjusted sensitivities S2

	Diagnostic Performance	
ALG #	Sensitivity S2 (%)	Specificity (%)
15.1	52.90	95.14
15	53.60	94.32
11.2	52.60	94.05
13	50.90	95.00
7	45.20	98.38
9	43.90	98.38
11.1	52.60	93.37
12.1	52.80	93.38
4.1	55.10	91.89
8.1	45.80	96.76
mean	**50.54**	**95.07**
SD	4.01	2.17
lower limit 95%CI	*48.06*	*93.72*
upper limit 95%CI	*53.02*	*96.41*

Notifications of 2008				
Notifications with Inno-Lia; N=661				All; N=763
N ruled older	N ruled incident	N estimated incident	IIR-P	Total N estimated incident
521	140	225	0.340	259
520	141	216	0.327	249
528	133	201	0.304	232
535	126	203	0.306	234
555	106	219	0.331	252
561	100	211	0.320	244
528	133	194	0.293	224
525	136	200	0.302	231
519	142	188	0.285	217
525	136	269	0.407	311
	mean	**212**	**0.321**	**245**
	SD	23	0.035	27
	lower limit 95%CI	*198*	*0.300*	*229*
	upper limit 95%CI	*227*	*0.343*	*262*

Notifications of 2009					
Notifications with Inno-Lia; N=599					All; N=649
N ruled older	N ruled incident	N estimated incident	IIR	IIR, in % of baseline	Total N estimated incident
480	119	187	0.312	92.0	203
477	122	184	0.306	93.8	199
478	121	183	0.305	100.6	198
493	106	166	0.277	90.3	180
517	82	166	0.277	83.7	180
524	75	154	0.258	80.7	167
478	121	177	0.295	100.6	192
477	122	178	0.298	98.5	193
467	132	178	0.296	104.1	192
476	123	243	0.406	99.8	264
	mean	**182**	**0.303**	**94.4**	**197**
	SD	24	0.040	7.8	26
	lower limit 95%CI	*167*	*0.278*	*89.6*	*181*
	upper limit 95%CI	*196*	*0.328*	*99.2*	*213*

Notifications of 2010					
Notifications with Inno-Lia; N=587					All; N=597
N ruled older	N ruled incident	N estimated incident	IIR	IIR, in % of baseline	Total N estimated incident
480	107	163	0.278	81.9	166
477	110	160	0.273	83.4	163
485	102	144	0.245	80.6	146
492	95	143	0.244	79.5	145
517	70	139	0.236	71.5	141
521	66	134	0.228	71.2	136
485	102	137	0.234	79.7	140
482	105	143	0.244	80.7	146
462	125	165	0.281	98.6	168
480	107	207	0.352	86.5	210
	mean	**153**	**0.261**	**81.4**	**156**
	SD	22	0.037	7.7	22
	lower limit 95%CI	*140*	*0.238*	*76.6*	*142*
	upper limit 95%CI	*167*	*0.284*	*86.1*	*170*

Notifications of 2011					
Notifications with Inno-Lia; N=549					All; N=557
N ruled older	N ruled incident	N estimated incident	IIR	IIR, in % of baseline	Total N estimated incident
462	87	126	0.229	67.3	127
456	93	129	0.235	71.9	131
455	94	131	0.239	78.8	133
468	81	117	0.213	69.4	118
485	64	126	0.230	69.6	128
491	58	116	0.212	66.2	118
455	94	125	0.228	77.8	127
453	96	129	0.235	77.9	131
451	98	114	0.207	72.8	115
450	99	191	0.348	85.3	194
	mean	**130**	**0.238**	**73.7**	**132**
	SD	22	0.040	6.1	22
	lower limit 95%CI	*117*	*0.213*	*69.9*	*118*
	upper limit 95%CI	*144*	*0.263*	*77.5*	*146*

C. Calculation of performance-based incident infection rate (IIR-P) utilizing the adjusted sensitivities S3

	Diagnostic Performance	
ALG #	Sensitivity S3 (%)	Specificity (%)
15.1	39.60	95.14
15	40.20	94.32
11.2	39.20	94.05
13	37.70	95.00
7	32.40	98.38
9	31.40	98.38
11.1	39.20	93.37
12.1	39.30	93.38
4.1	42.10	91.89
8.1	33.10	96.76
mean	**37.42**	**95.07**
SD	3.72	2.17
lower limit 95%CI	*35.12*	*93.72*
upper limit 95%CI	*39.72*	*96.41*

Notifications of 2008				
Notifications with Inno-Lia; N=661				All; N=763
N ruled older	N ruled incident	N estimated incident	IIR-P	Total N estimated incident
521	140	311	0.470	358
520	141	300	0.453	346
528	133	282	0.426	325
535	126	284	0.430	328
555	106	310	0.468	357
561	100	300	0.454	346
528	133	274	0.414	316
525	136	282	0.427	326
519	142	260	0.393	300
525	136	384	0.581	443
	mean	**299**	**0.452**	**345**
	SD	34	0.051	39
	lower limit 95%CI	*278*	*0.420*	*320*
	upper limit 95%CI	*320*	*0.483*	*369*

Notifications of 2009					
Notifications with Inno-Lia; N=599					All; N=649
N ruled older	N ruled incident	N estimated incident	IIR	IIR, in % of baseline	Total N estimated incident
480	119	259	0.432	92.0	280
477	122	255	0.425	93.8	276
478	121	257	0.429	100.6	278
493	106	233	0.388	90.3	252
517	82	235	0.392	83.7	254
524	75	219	0.366	80.7	238
478	121	250	0.417	100.6	270
477	122	252	0.421	98.5	273
467	132	245	0.410	104.1	266
476	123	347	0.579	99.8	376
	mean	**255**	**0.426**	**94.4**	**276**
	SD	35	0.058	7.8	38
	lower limit 95%CI	*234*	*0.390*	*89.6*	*253*
	upper limit 95%CI	*277*	*0.462*	*99.2*	*300*

Notifications of 2010					
Notifications with Inno-Lia; N=587					All; N=597
N ruled older	N ruled incident	N estimated incident	IIR	IIR, in % of baseline	Total N estimated incident
480	107	226	0.385	81.9	230
477	110	222	0.378	83.4	226
485	102	202	0.344	80.6	205
492	95	201	0.342	79.5	204
517	70	197	0.335	71.5	200
521	66	190	0.323	71.2	193
485	102	194	0.330	79.7	197
482	105	202	0.345	80.7	206
462	125	228	0.388	98.6	232
480	107	295	0.502	86.5	300
	mean	**216**	**0.367**	**81.4**	**219**
	SD	31	0.053	7.7	32
	lower limit 95%CI	*196*	*0.334*	*76.6*	*200*
	upper limit 95%CI	*235*	*0.400*	*86.1*	*239*

Notifications of 2011					
Notifications with Inno-Lia; N=549					All; N=557
N ruled older	N ruled incident	N estimated incident	IIR	IIR, in % of baseline	Total N estimated incident
462	87	174	0.316	67.3	176
456	93	179	0.326	71.9	182
455	94	184	0.336	78.8	187
468	81	164	0.298	69.4	166
485	64	179	0.326	69.6	182
491	58	165	0.300	66.2	167
455	94	177	0.322	77.8	179
453	96	183	0.333	77.9	185
451	98	157	0.287	72.8	160
450	99	272	0.495	85.3	276
	mean	**183**	**0.334**	**73.7**	**186**
	SD	32	0.059	6.1	33
	lower limit 95%CI	*163*	*0.297*	*69.9*	*166*
	upper limit 95%CI	*203*	*0.371*	*77.5*	*206*

Notifications of 2012					
Notifications with Inno-Lia; N=643					All; N=643
N ruled older	N ruled incident	N estimated incident	IIR	IIR, in % of baseline	Total N estimated incident
551	92	225	0.349	57.9	225
546	97	225	0.349	60.1	225
554	89	199	0.310	55.7	199
561	82	199	0.310	55.1	199
574	69	255	0.396	63.2	255
581	62	234	0.363	59.4	234
554	89	187	0.291	53.5	187
554	89	187	0.291	51.7	187
542	101	184	0.287	56.8	184
553	90	315	0.490	62.1	315
mean		**221**	**0.344**	**57.5**	**221**
SD		40	0.063	3.7	40
lower limit 95%CI		*196*	*0.305*	*55.3*	*196*
upper limit 95%CI		*246*	*0.383*	*59.8*	*246*

Notifications of 2013					
Notifications with Inno-Lia; N=597					All; N=600
N ruled older	N ruled incident	N estimated incident	IIR	IIR, in % of baseline	Total N estimated incident
519	78	181	0.303	50.3	182
516	81	175	0.293	50.4	176
519	78	167	0.280	50.2	168
527	70	161	0.269	47.8	161
540	57	206	0.345	55.0	207
545	52	192	0.321	52.5	193
519	78	155	0.260	47.7	156
518	79	159	0.267	47.4	160
510	87	146	0.244	48.3	146
515	82	285	0.478	60.5	287
mean		**183**	**0.306**	**51.0**	**184**
SD		40	0.068	4.1	41
lower limit 95%CI		*158*	*0.264*	*48.5*	*158*
upper limit 95%CI		*208*	*0.348*	*53.6*	*209*

All 3809 notifications, 2008–2013	
Total n estimated incident	Mean IIR
1749	0.459
1721	0.452
1668	0.438
1604	0.421
1792	0.471
1699	0.446
1609	0.422
1653	0.434
1559	0.409
2498	0.656
**1755**	**0.461**
270	0.071
1588	*0.417*
1923	*0.505*

Notifications of 2012					
Notifications with Inno-Lia; N=643					All; N=643
N ruled older	N ruled incident	N estimated incident	IIR	IIR, in % of baseline	Total N estimated incident
551	92	126	0.197	57.9	126
546	97	126	0.196	60.1	126
554	89	109	0.169	55.7	109
561	82	109	0.169	55.1	109
574	69	134	0.209	63.2	134
581	62	122	0.190	59.4	122
554	89	101	0.157	53.5	101
554	89	101	0.156	51.7	101
542	101	104	0.162	56.8	104
553	90	163	0.253	62.1	163
mean		**119**	**0.186**	**57.5**	**119**
SD		19	0.030	3.7	19
lower limit 95%CI		*107*	*0.167*	*55.3*	*107*
upper limit 95%CI		*131*	*0.204*	*59.8*	*131*

Notifications of 2013					
Notifications with Inno-Lia; N=597					All; N=600
N ruled older	N ruled incident	N estimated incident	IIR	IIR, in % of baseline	Total N estimated incident
519	78	102	0.171	50.3	102
516	81	98	0.165	50.4	99
519	78	91	0.153	50.2	92
527	70	87	0.147	47.8	88
540	57	109	0.182	55.0	109
545	52	100	0.168	52.5	101
519	78	84	0.140	47.7	84
518	79	85	0.143	47.4	86
510	87	82	0.138	48.3	83
515	82	147	0.247	60.5	148
mean		**99**	**0.165**	**51.0**	**99**
SD		19	0.032	4.1	19
lower limit 95%CI		*87*	*0.145*	*48.5*	*87*
upper limit 95%CI		*111*	*0.185*	*53.6*	*111*

All 3809 notifications, 2008–2013	
Total n estimated incident	Mean IIR
984	0.258
967	0.254
910	0.239
874	0.229
945	0.248
887	0.233
867	0.228
887	0.233
879	0.231
1289	0.338
**949**	**0.249**
126	0.033
871	*0.229*
1027	*0.270*

Notifications of 2012					
Notifications with Inno-Lia; N=643					All; N=643
N ruled older	N ruled incident	N estimated incident	IIR	IIR, in % of baseline	Total N estimated incident
551	92	175	0.272	57.9	175
546	97	175	0.272	60.1	175
554	89	153	0.237	55.7	153
561	82	152	0.237	55.1	152
574	69	190	0.296	63.2	190
581	62	173	0.269	59.4	173
554	89	142	0.221	53.5	142
554	89	142	0.221	51.7	142
542	101	144	0.224	56.8	144
553	90	232	0.360	62.1	232
mean		**168**	**0.261**	**57.5**	**168**
SD		28	0.044	3.7	28
lower limit 95%CI		*150*	*0.234*	*55.3*	*150*
upper limit 95%CI		*185*	*0.288*	*59.8*	*185*

Notifications of 2013					
Notifications with Inno-Lia; N=597					All; N=600
N ruled older	N ruled incident	N estimated incident	IIR	IIR, in % of baseline	Total N estimated incident
519	78	141	0.236	50.3	142
516	81	136	0.229	50.4	137
519	78	128	0.214	50.2	128
527	70	123	0.206	47.8	123
540	57	154	0.258	55.0	155
545	52	142	0.238	52.5	143
519	78	118	0.198	47.7	119
518	79	121	0.202	47.4	121
510	87	114	0.190	48.3	114
515	82	210	0.351	60.5	211
mean		**139**	**0.232**	**51.0**	**139**
SD		28	0.047	4.1	28
lower limit 95%CI		*121*	*0.203*	*48.5*	*122*
upper limit 95%CI		*156*	*0.261*	*53.6*	*157*

All 3809 notifications, 2008–2013	
Total n estimated incident	Mean IIR
1361	0.357
1342	0.352
1277	0.335
1226	0.322
1338	0.351
1260	0.331
1224	0.321
1253	0.329
1215	0.319
1837	0.482
**1333**	**0.350**
185	0.048
1219	*0.320*
1448	*0.380*
